# Radiomics in Lung Cancer Imaging: A Narrative Review of Current Evidence

**DOI:** 10.3390/jimaging12070287

**Published:** 2026-06-29

**Authors:** Andrea Lastrucci, Nicola Iosca, Edoardo Cavigli, Diletta Cozzi, Angelo Barra, Yannick Wandael, Cosimo Nardi, Renzo Ricci, Vittorio Miele, Daniele Giansanti

**Affiliations:** 1 Department of Allied Health Professions, Azienda Ospedaliero-Universitaria Careggi, 50134 Florence, Italyioscan@aou-careggi.toscana.it (N.I.); barraa@aou-careggi.toscana.it (A.B.); wandaely@aou-careggi.toscana.it (Y.W.); riccire@aou-careggi.toscana.it (R.R.); 2 Department of Emergency Radiology, Careggi University Hospital, L.Go Brambilla 3, 50123 Florence, Italy; caviglie@aou-careggi.toscana.it (E.C.); cozzid@aou-careggi.toscana.it (D.C.); vmiele@sirm.org (V.M.); 3 Department of Experimental and Clinical Biomedical Sciences “Mario Serio”, University of Florence, 50134 Florence, Italy; cosimo.nardi@unifi.it; 4 Centre IATIS, Istituto Superiore di Sanità, 00161 Rome, Italy

**Keywords:** radiomics, lung cancer, computed tomography, artificial intelligence, radiogenomics, radiology

## Abstract

Background: Lung cancer remains the leading cause of cancer-related mortality worldwide, and early diagnosis and accurate disease stratification are still major clinical challenges. Radiomics has emerged as a quantitative imaging approach that extracts high-dimensional features from radiological imaging, with applications in diagnosis, prognosis, radio genomics, and assessment of treatment response. However, its clinical translation is still limited by methodological heterogeneity and a lack of standardization. Aim: This narrative review synthesizes evidence from systematic reviews and meta-analyses on radiomics in thoracic imaging for lung cancer, focusing on clinical applications, methodological limitations, and translational challenges. Methods: A structured search was conducted in PubMed and Scopus using predefined keywords related to radiomics, lung cancer, and imaging modalities. Only peer-reviewed systematic reviews and meta-analyses published in English were included. In total, 27 studies were selected and synthesized using a structured narrative approach guided by the ANDJ checklist. A differential integrative framework was adopted to connect evidence from systematic reviews and meta-analyses with primary empirical studies and policy documents through an intermediate layer of translational recommendations, ensuring a multi-level and interpretation-driven synthesis. Results: Radiomics demonstrated consistent potential across multiple clinical domains, including lesion classification, histological differentiation, molecular profiling, prognostic stratification, and prediction of treatment response. Machine learning and deep learning approaches frequently improved predictive performance. However, key limitations were identified, including heterogeneity in imaging protocols, lack of external validation, small single-centre datasets, and limited reproducibility of radiomic features. Conclusions: Radiomics in lung cancer imaging shows strong clinical potential but remains constrained by methodological and translational barriers. Future progress will depend on standardization, external validation, multimodal data integration, and improved interpretability, alongside alignment with regulatory and clinical implementation frameworks.

## 1. Introduction

Lung cancer remains a major global health challenge and the leading cause of cancer-related mortality worldwide, accounting for approximately 1.8 million deaths annually and nearly one-fifth of all cancer deaths [1]. Despite advances in targeted therapies and immunotherapy, survival rates remain poor, largely due to late-stage diagnosis, when curative treatment options are limited and prognosis is unfavorable [2]. Screening with low-dose computed tomography (LDCT) has demonstrated significant mortality reductions in high-risk populations, although its implementation remains inconsistent across healthcare systems [3]. The complexity of lung cancer management is further influenced by heterogeneous tumor biology, overlapping clinical presentations, and diagnostic delays, highlighting the need for improved biomarkers and quantitative tools to enable earlier detection, risk stratification, and personalized treatment strategies [2,4].

Medical imaging plays a pivotal role in the diagnosis, staging, and management of lung cancer. LDCT is the cornerstone of screening, while nuclear medicine imaging provides complementary metabolic information for lesion characterization, staging, and response assessment [5,6]. Magnetic resonance imaging (MRI) has a more limited role in primary lung lesion detection but is valuable for evaluating mediastinal and chest wall invasion, as well as for detecting brain metastases without ionizing radiation [7]. However, conventional imaging interpretation relies largely on qualitative assessment, which is subject to interobserver variability and may fail to capture subtle tumor heterogeneity [8]. These limitations underscore the need for objective and reproducible quantitative imaging biomarkers.

Radiomics has emerged as a promising approach to extract high-dimensional quantitative features from medical images, including intensity, shape, and texture descriptors that capture tumor heterogeneity beyond visual perception [9]. The radiomics workflow typically involves lesion segmentation, feature extraction, and integration with statistical or machine learning models to generate predictive signatures [9,10]. In lung cancer, radiomics has shown potential in prognostic stratification, treatment response prediction, and identification of molecular characteristics, supporting non-invasive precision medicine approaches [10,11,12].

Despite this growing interest, several methodological challenges hinder clinical translation. Radiomic features are highly sensitive to variations in imaging acquisition, reconstruction algorithms, segmentation methods, and preprocessing techniques, leading to limited reproducibility and comparability across studies [13,14,15]. Additionally, most studies are based on small, single-center cohorts with limited external validation, restricting generalizability [16,17]. These issues highlight the need for standardized workflows, larger multicenter datasets, and more rigorous validation strategies.

Efforts to improve standardization and reproducibility, such as the Image Biomarker Standardisation Initiative (IBSI) and resources from the Radiological Society of North America (RSNA), reflect the growing recognition of these challenges [18,19]. However, methodological heterogeneity and variability in study quality persist across the literature.

To provide a structured synthesis of the field, this narrative review addresses the following key questions:

**Q1:**  *Which radiomic features and radiological (CT-MRI) imaging modalities demonstrate the highest reproducibility and robustness in lung cancer imaging?*

**Q2:**  *What emerging themes are evident in systematic reviews, including novel applications and predictive models?*

**Q3:**  *What methodological strengths and limitations are reported, particularly regarding feature extraction, acquisition protocols, and validation strategies?*

**Q4:**  *How have radiomics approaches contributed to clinical outcomes such as prognosis and treatment response?*

**Q5:**  *What gaps remain, and what opportunities exist for advancing radiomics toward clinical implementation?*

### Aim of the Review

The aim of this narrative review is to provide an overview of current evidence from systematic reviews and meta-analyses on radiomics in thoracic imaging for lung cancer, with a critical discussion of methodological considerations, clinical applications, and key challenges for future research and clinical translation.

## 2. Study Design

This narrative review provides an interpretative synthesis of the literature on radiomics applications in thoracic imaging for lung cancer. A narrative approach was adopted rather than a systematic review because of the inherently heterogeneous and rapidly evolving nature of radiomics research, which spans diverse methodological pipelines, feature extraction strategies, validation frameworks, and levels of clinical maturity. In this context, a systematic review design, with its predefined and rigid methodological constraints and formalized quality assessment procedures, would risk oversimplifying the complexity and variability of the available evidence, potentially limiting the ability to capture broader clinical and translational insights.

Instead, a narrative approach allows for a more flexible and context-sensitive integration of evidence across different levels of synthesis, particularly where findings from systematic reviews and meta-analyses need to be interpreted in relation to clinical applicability and translational development.

This narrative review focused on peer-reviewed systematic reviews and meta-analyses on radiomics applications in thoracic imaging for lung cancer, with particular attention to clinical applications, methodological limitations, and translational challenges. The literature search was conducted in Scopus and PubMed, two major biomedical databases widely used in radiology, oncology, and medical imaging research, selected to ensure broad and complementary coverage of the peer-reviewed literature.

A targeted literature search strategy was developed using a combination of controlled vocabulary and free-text terms related to radiomics, lung cancer, and imaging modalities. The search strategy included the following terms:

*(radiomics[Title/Abstract] OR radiomic[Title/Abstract] OR “radiomic features”[Title/Abstract] OR “texture analysis”[Title/Abstract])*

*AND*

(“lung cancer”[Title/Abstract] OR “lung carcinoma”[Title/Abstract] OR “lung neoplasm*”[Title/Abstract] OR “pulmonary tumor*”[Title/Abstract] OR NSCLC[Title/Abstract] OR “non small cell lung cancer”[Title/Abstract] OR SCLC[Title/Abstract])

*AND*

*(“computed tomography”[Title/Abstract] OR CT[Title/Abstract] OR “magnetic resonance imaging”[Title/Abstract] OR MRI[Title/Abstract])*

No temporal restrictions were applied in order to capture the full breadth and evolution of radiomics research in lung cancer imaging. Although no formal temporal restrictions were applied, priority was given to more recent studies that have incorporated or updated evidence from earlier literature, reflecting the progressive consolidation of radiomics research in lung cancer imaging.

Only peer-reviewed articles published in the English language were considered for inclusion.

The selection of studies was guided by a focus on systematic reviews and meta-analyses, which represent high-level synthesized evidence in radiomics research and allow the identification of consistent findings, methodological limitations, and persisting gaps across multiple clinical domains. The evidence base was therefore oriented toward studies addressing thoracic imaging modalities, including CT and MRI, and reporting clinically relevant applications such as diagnosis, histological characterization, radiogenomics, prognostic stratification, or treatment response assessment in human lung cancer populations, including NSCLC, SCLC, and other pulmonary malignancies. Primary research articles, non-peer-reviewed publications, conference abstracts, editorials, and commentaries were not included, nor were narrative reviews without explicit systematic methodology, purely theoretical or computational radiomics studies without a clinical imaging context, studies not focused on lung cancer or thoracic imaging modalities, or articles not available in full text or not published in English.

To enhance the interpretative structure of the synthesis and ensure coherence across heterogeneous evidence sources, the analysis was organized according to a multi-level integrative framework. Specifically, findings from systematic reviews and meta-analyses were used to derive a set of emerging methodological and translational recommendations (R1–R6), which subsequently served as an interpretative scaffold for the analysis of primary empirical studies. In parallel, international policy, regulatory, and standardization documents were incorporated as an additional layer of evidence to contextualize translational feasibility, clinical implementation, and governance constraints. This hierarchical structure allowed for a structured integration between evidence synthesis, empirical validation, and implementation frameworks within a unified narrative approach.

The synthesis followed a narrative approach, enabling interpretative integration across heterogeneous domains of application, including diagnosis, radiogenomic analysis, prognostic stratification, and treatment response assessment. Methodological transparency and reporting quality were guided by the ANDJ Narrative Review Checklist [20], used as a reporting framework to ensure clarity and completeness without implying a systematic review design.

## 3. Results

This section is organized into three complementary levels of synthesis.

Section 3.1 provides a descriptive overview and sketch of the included systematic reviews, outlining their main characteristics and the distribution of evidence across clinical application domains in lung cancer.

Section 3.2 presents a *thematic synthesis* of radiomics applications, structured according to the principal clinical domains, including diagnostic applications, radiogenomics, prognostic stratification, treatment response, and the role of artificial intelligence.

Section 3.3 develops a multilevel synthesis integrating clinical applications, methodological variability, and algorithmic frameworks, and is structured into four sub-sections: Section 3.3.1 focuses on radiomics applications and AI modelling in lung cancer, Section 3.3.2 compares methodological approaches across studies, Section 3.3.3 analyses algorithmic frameworks and key technical constraints, and Section 3.3.4 provides an integrated interpretative synthesis of the overall evidence.

### 3.1. Study Selection and Characteristics

A total of 27 systematic reviews and meta-analyses [21,22,23,24,25,26,27,28,29,30,31,32,33,34,35,36,37,38,39,40,41,42,43,44,45,46,47] met the predefined inclusion criteria and were included in the final synthesis.

The included studies encompass a broad range of radiomics applications in lung cancer, spanning diagnostic, radiogenomic, prognostic, treatment response, and methodological domains. Table 1 reports the classification of the included systematic reviews according to their primary application domain, providing a structured overview of the evidence base.

A brief sketch of each included study is provided in the Supplementary Materials to ensure readability and reduce redundancy within the main manuscript.

Overall, the included evidence reflects a heterogeneous but rapidly expanding field, in which radiomics and artificial intelligence (AI) are increasingly investigated for multiple clinical applications, ranging from lesion characterization and molecular prediction to outcome stratification and treatment monitoring.

### 3.2. Integrated Thematic Synthesis

Across the included evidence, radiomics is consistently framed as an extension of medical imaging that enables the extraction of high-dimensional quantitative information not directly accessible through conventional visual assessment. When integrated with artificial intelligence (AI), it progressively evolves from a feature engineering approach into a broader computational framework for clinical decision support in lung cancer, with applications spanning diagnosis, radiogenomics, prognostic stratification, and treatment response evaluation.

Rather than representing isolated technical applications, the literature suggests a gradual convergence toward a more integrated paradigm, in which imaging-derived features are increasingly interpreted as proxies for tumour phenotype, underlying molecular alterations, and clinically relevant outcomes. However, this convergence is still partial, as the maturity and robustness of evidence differ substantially across domains and study designs.

#### 3.2.1. Diagnostic Applications

Diagnostic applications constitute one of the most consolidated areas of radiomics research in lung cancer. Across studies, radiomics-based models have demonstrated the ability to support lesion characterization, particularly in distinguishing benign from malignant pulmonary nodules and in classifying histological subtypes. This diagnostic potential is especially evident when radiomics is combined with advanced imaging modalities such as dual-energy CT or with machine learning classifiers, which consistently enhance discriminatory performance [22,24,41].

Beyond conventional diagnostic tasks, radiomics has also been explored for the identification of more subtle and clinically relevant pathological features, including tumour invasiveness and specific histopathological patterns such as STAS. These features are particularly relevant because they are difficult to assess reliably through standard radiological interpretation alone, suggesting that radiomics may contribute to a deeper, phenotype-oriented characterization of lung cancer [28,31,36].

Despite these encouraging findings, the diagnostic literature remains affected by substantial methodological variability. Differences in imaging acquisition protocols, reconstruction parameters, segmentation strategies, and feature extraction pipelines are consistently reported across studies. This heterogeneity not only limits reproducibility but also complicates the establishment of stable diagnostic biomarkers that can be generalized across clinical settings.

#### 3.2.2. Radiogenomics and Molecular Prediction

A second major thematic area concerns radiogenomics, where radiomics features are used as non-invasive surrogates to infer underlying molecular and genetic characteristics of tumours. Across the included evidence, radiomics-based models have been associated with several clinically relevant biomarkers, including EGFR, ALK, and KRAS mutations, as well as PD-L1 expression and Ki-67 proliferation index [21,27,32,38,42].

In this context, machine learning and deep learning approaches appear to play a crucial role in improving predictive performance, particularly when dealing with complex, high-dimensional imaging data. These methods allow more flexible modelling of non-linear relationships between imaging phenotypes and molecular alterations, supporting the hypothesis that radiological features may partially encode genomic and immunological information [30,39].

However, despite these promising signals, the translational value of radiogenomics remains limited. Most studies rely on retrospective single- or small-cohort datasets, and only a minority include robust external validation. In addition, the absence of standardized radiomics workflows—including feature definition, selection strategies, and modelling pipelines—introduces further variability, reducing the reliability and reproducibility of reported associations.

#### 3.2.3. Prognostic Stratification

Radiomics is also extensively applied in prognostic modelling, particularly for the stratification of lung cancer patients according to survival outcomes and treatment response trajectories. Across studies, radiomics features—often integrated with clinical variables—demonstrate consistent ability to discriminate between different risk groups in terms of overall survival and progression-free survival, especially in advanced disease and in patients undergoing immunotherapy or targeted therapies [29,40,43,44].

An emerging evolution of this approach is represented by dynamic and longitudinal modelling strategies, such as delta radiomics, which capture temporal changes in tumour characteristics across serial imaging examinations. These approaches appear to provide additional prognostic information compared to single-timepoint analyses, suggesting that tumour evolution may be as informative as baseline phenotype [40].

Nevertheless, the prognostic evidence base is characterized by considerable heterogeneity in study design, patient populations, and analytical strategies. Most studies remain retrospective, and external validation is often limited or absent, which collectively constrains the clinical robustness and generalizability of prognostic models.

#### 3.2.4. Treatment Response and Monitoring

Another relevant application domain is treatment response assessment, where radiomics is used to predict and monitor therapeutic effects, particularly in patients undergoing chemoimmunotherapy or radiotherapy. In this setting, radiomics provides a non-invasive means of capturing early imaging changes associated with treatment response, potentially enabling earlier identification of responders and non-responders [23,25].

This capability is particularly relevant in the context of precision oncology, where treatment adaptation based on early response evaluation could improve patient stratification and therapeutic efficiency. However, the reliability of these approaches is influenced by variability in imaging quality, acquisition protocols, and modality-specific limitations, particularly in cone-beam CT-based studies used during radiotherapy.

As a result, while treatment response modelling represents one of the most clinically attractive applications of radiomics, its current implementation remains limited by technical and methodological inconsistencies.

#### 3.2.5. Role of Artificial Intelligence in Radiomics

Artificial intelligence represents a key enabling layer across all radiomics applications. Machine learning and deep learning approaches contribute primarily to improving feature selection, reducing dimensionality, and modelling complex interactions within high-dimensional imaging data.

Beyond these technical contributions, AI also facilitates the integration of multimodal data sources, combining radiomics features with clinical and genomic information to enhance predictive accuracy and improve biological interpretability [30,39]. In particular, deep learning approaches have demonstrated strong performance in several predictive tasks, often outperforming traditional machine learning pipelines in complex classification problems.

However, these advances are accompanied by important limitations. Many AI-based radiomics models are developed on limited or single-institution datasets, which restricts generalizability. Furthermore, performance degradation during external validation remains a recurring issue, often reflecting domain shift between imaging protocols or populations. Finally, the interpretability of many deep learning models remains limited, raising concerns regarding clinical trust and implementation.

*Integrated interpretation*

Taken together, the evidence portrays radiomics as a highly promising but still evolving framework for quantitative imaging in lung cancer. Its integration with artificial intelligence significantly expands its potential across multiple clinical domains, yet the field remains constrained by methodological heterogeneity, lack of standardization, and limited external validation. These factors collectively slow its transition from a predominantly research-driven tool toward routine clinical application.

### 3.3. Multilevel Synthesis of Radiomics and Artificial Intelligence Evidence in Lung Cancer

This section provides a multilevel synthesis of radiomics and artificial intelligence evidence in lung cancer, integrating findings across clinical application domains, methodological designs, and algorithmic modelling strategies.

Across the included studies, radiomics and AI are applied to diagnostic, radiogenomic, prognostic, and treatment-related tasks, with a predominant reliance on machine learning approaches and an emerging role of deep learning.

The synthesis also highlights substantial methodological variability across imaging pipelines, feature extraction strategies, validation designs, and outcome definitions, which limits direct comparability between studies.

Finally, the analysis addresses key algorithmic constraints, including issues of generalizability, interpretability, and external validation, which collectively affect the robustness and clinical transferability of radiomics-based AI models.

#### 3.3.1. Integrated Synthesis of Radiomics Applications and AI Modelling in Lung Cancer Treatment

The synthesis of the included systematic reviews highlights a structured distribution of radiomics applications across multiple clinical domains in lung cancer. The overview reported in Table S1 in the Supplementary Materials summarizes, for each study, the primary radiomics contribution, the reported role of artificial intelligence (AI), and the main methodological opportunities identified by the authors.

Across diagnostic applications, radiomics is consistently reported as a tool for improving lesion characterization and histological classification. In particular, dual-energy CT-based approaches are associated with high diagnostic performance [22], while machine learning-based frameworks are frequently applied to classification tasks such as histological subtype identification and malignancy assessment [24,41]. Additional diagnostic applications include the detection of specific pathological features, such as STAS, where classification models are commonly employed [28,31]. These applications are recurrent across Table S1, which consistently reports classification-oriented AI strategies in diagnostic settings.

In radiogenomic applications, radiomics is mainly used for the prediction of molecular and genetic biomarkers, including EGFR, ALK, KRAS mutations, PD-L1 expression, and Ki-67 proliferation index [21,27,32,38,42]. According to Table S1, machine learning approaches are predominantly used for feature integration and classification tasks, while deep learning is reported as improving predictive performance in selected studies [39]. However, most studies highlight the need for improved generalizability, external validation, and standardization of analytical pipelines.

Prognostic applications represent another major domain, where radiomics is used for survival stratification, including overall survival and progression-free survival in NSCLC patients [29,40,43,44]. Table S1 shows that these models are primarily based on texture-derived features and machine learning approaches. In some cases, advanced modelling strategies such as delta radiomics and longitudinal imaging analysis are used to capture temporal changes [40]. Across studies, recurrent limitations include retrospective study designs and the need for external validation.

Treatment-related applications include prediction of response to therapy and imaging-based monitoring. Radiomics has been applied to assess pathological response to chemoimmunotherapy [23] and to monitor treatment effects using CBCT imaging during radiotherapy [25]. In these contexts, AI is reported as supporting longitudinal modelling and improving predictive performance. However, variability in imaging acquisition protocols and methodological heterogeneity are consistently reported as limiting factors.

Overall, Table S1 indicates that machine learning remains the predominant AI approach across most radiomics applications, while deep learning is reported in a smaller subset of studies, mainly associated with improved predictive performance in specific tasks. Across all domains, the most frequently reported methodological needs include standardization of imaging protocols, improvement of external validation, and access to larger multicenter datasets.

#### 3.3.2. Methodological Comparison Across Included Evidence

Across the included systematic reviews, substantial methodological variability was observed, reflecting the intrinsic heterogeneity of radiomics research in lung cancer. Rather than arising from differences in clinical domains alone, this variability is strongly driven by differences in study design, imaging pipelines, and model development strategies, which collectively limit cross-study comparability and reproducibility.

A first relevant dimension of heterogeneity concerns the imaging input and feature extraction strategies. Most studies rely on CT-based radiomics, yet acquisition protocols, reconstruction parameters, and segmentation approaches differ widely across investigations. This is particularly evident in applications involving dual-energy CT, CBCT, or longitudinal imaging, where variability in image quality and preprocessing pipelines introduces significant methodological noise [22,25,40]. Such inconsistencies directly affect feature stability and may partially explain the variability in reported predictive performance across studies addressing similar clinical endpoints.

A second level of methodological divergence relates to model development strategies. While earlier studies predominantly employed classical machine learning approaches based on handcrafted radiomic features, more recent evidence increasingly integrates deep learning frameworks or hybrid radiomics–AI pipelines. This evolution is evident in studies focusing on molecular prediction and histological classification, where deep learning approaches are often reported to outperform traditional radiomics-based models [24,30,39]. However, this gain in performance is frequently accompanied by reduced interpretability and limited methodological transparency, particularly in end-to-end deep learning models.

A further relevant aspect concerns validation strategies. Across the included evidence, internal validation is common, whereas external validation remains limited or inconsistently reported. Several studies explicitly highlight performance drops when models are tested on independent datasets, suggesting a strong influence of dataset-specific characteristics and potential overfitting [23,30,33]. This issue is particularly relevant in radiogenomics and prognostic applications, where generalizability across institutions is critical for clinical translation.

In addition, heterogeneity in ground truth definition and outcome measurement further complicates methodological comparison. Diagnostic endpoints (e.g., malignancy, histological subtype, STAS) are generally more standardized, whereas molecular and prognostic endpoints (e.g., PD-L1 expression, survival outcomes, treatment response) are defined using variable thresholds, assays, and follow-up strategies across studies [27,29,38,43]. This variability limits the possibility of direct quantitative comparison between models addressing similar clinical questions.

Overall, the methodological synthesis highlights that differences in performance across studies are not solely attributable to model choice, but rather emerge from a combination of imaging heterogeneity, feature engineering strategies, validation design, and outcome definition. These factors collectively represent a key barrier to reproducibility and clinical translation, and partially explain the variability observed across the evidence summarized in Table S1 of the Supplementary Materials.

#### 3.3.3. Algorithmic Frameworks, Generalizability, and Technical Constraints in Radiomics-Based AI Models

Across the included evidence, radiomics-based artificial intelligence systems are predominantly built on a relatively limited set of algorithmic paradigms, primarily consisting of traditional machine learning models trained on handcrafted imaging features, and more recently, deep learning architectures capable of end-to-end feature learning. This duality reflects an ongoing transition in the field, where classical radiomics pipelines coexist with emerging data-driven approaches, without yet achieving methodological convergence.

Classical radiomics approaches, largely represented across earlier and many intermediate studies, rely on predefined feature extraction followed by supervised machine learning classification or regression models. These pipelines are particularly evident in diagnostic and prognostic applications, where structured feature sets derived from CT imaging are used to predict outcomes such as malignancy, histological subtype, or survival endpoints [22,24,41,43]. While these approaches offer a degree of interpretability, their performance is highly dependent on feature selection strategies and preprocessing choices, which are known to vary significantly across studies.

In contrast, deep learning-based approaches, increasingly reported in more recent studies, reduce dependence on handcrafted features by learning hierarchical representations directly from imaging data. These models are particularly prominent in radiogenomic and response prediction tasks, where they are often reported to outperform traditional radiomics pipelines [30,39]. However, this performance advantage is not consistent across all studies and is frequently accompanied by reduced transparency and limited interpretability, particularly in end-to-end architectures where feature attribution remains challenging.

A central methodological constraint emerging from the synthesis is the issue of generalizability. Across multiple application domains, including molecular prediction and prognostic modelling, a recurrent pattern is the reliance on single-centre or retrospectively curated datasets, with limited external validation [23,30,33,35]. This leads to a structural risk of domain shift, where models perform well under internal validation but exhibit reduced robustness when applied to independent cohorts. This issue is particularly critical in radiogenomics applications, where biological and imaging variability may further amplify dataset-specific bias.

Another key limitation lies in the sensitivity of radiomics pipelines to technical and pre-analytical factors. Variations in image acquisition (including CT, dual-energy CT, and CBCT), reconstruction parameters, and segmentation strategies introduce non-trivial variability in feature distributions [22,25,40]. As a consequence, even when similar machine learning architectures are used, model performance can diverge substantially due to upstream differences in data generation rather than algorithmic design itself.

Finally, the integration of multimodal data—combining radiomics with clinical and genomic information—represents an emerging direction across several included studies [21,33]. While such integration has been associated with improved predictive performance, it also increases model complexity and amplifies challenges related to harmonization, missing data handling, and reproducibility.

Overall, the algorithmic landscape of radiomics-based AI in lung cancer appears characterized by a tension between increasing model complexity and persistent limitations in standardization, interpretability, and external validity.

#### 3.3.4. Integrated Synthesis and Overarching Interpretative Framework

The synthesis of the included systematic reviews highlights a coherent yet highly heterogeneous body of evidence in which radiomics and artificial intelligence converge toward a shared goal: the extraction of clinically meaningful imaging biomarkers for lung cancer characterization. Despite the diversity of applications and methodological approaches, a set of recurrent cross-cutting patterns can be identified across diagnostic, radiogenomic, prognostic, and treatment-related domains.

A first overarching observation is the progressive convergence of radiomics applications toward a multi-dimensional clinical framework, in which imaging is no longer restricted to morphological assessment but increasingly reflects underlying molecular, pathological, and temporal disease characteristics. This is evident across studies addressing diagnostic classification [22,24,41], molecular prediction [21,27,32,38,42], prognostic stratification [29,40,43,44], and treatment response monitoring [23,25]. Taken together, these domains suggest a gradual transition from isolated predictive tasks toward integrated decision-support systems, although this transition remains largely exploratory.

A second key interpretative element concerns the asymmetry between technical advancement and methodological consolidation. While algorithmic sophistication has increased, particularly through the incorporation of deep learning and multimodal frameworks [30,33,39], this evolution has not been matched by equivalent progress in standardization and external validation. As a consequence, improvements in predictive performance often coexist with persistent uncertainty regarding reproducibility and cross-institutional robustness.

Third, the evidence consistently points to a central role of data quality and imaging harmonization as limiting factors that transcend specific clinical applications. Variability in acquisition protocols, reconstruction settings, and segmentation strategies emerges not as a secondary issue, but as a structural determinant of model instability across studies [22,25,40]. This suggests that methodological heterogeneity operates upstream of algorithmic performance and may partially explain inconsistencies observed even among studies addressing similar endpoints.

Finally, when considered collectively, the included evidence supports the interpretation that radiomics-based AI in lung cancer is currently positioned in a translational intermediate phase. On one hand, multiple studies demonstrate consistent proof-of-concept performance across different clinical scenarios; on the other hand, limitations in generalizability, transparency, and validation still prevent robust clinical integration. This duality is clearly reflected in the synthesis reported in Table S1 of the Supplementary Materials, which illustrates both the breadth of applications and the persistence of shared methodological constraints.

Overall, the integrated analysis suggests that future progress will depend less on incremental algorithmic refinement and more on the consolidation of shared standards, multicentre validation frameworks, and harmonized imaging pipelines capable of supporting reproducible and scalable radiomics-based AI systems.

## 4. Discussion

The discussion is structured into six interconnected sections that collectively aim to provide a comprehensive, multi-level interpretation of radiomics research in lung cancer imaging. Rather than following a purely linear or descriptive approach, the structure reflects the inherent complexity of the field, where evidence emerges from heterogeneous sources and must be interpreted across methodological, clinical, and translational dimensions. For this reason, the discussion progressively moves from consolidated evidence synthesis to interpretative frameworks, then to empirical validation, and finally to implementation-oriented and forward-looking considerations.

The first section (Section 4.1) presents the key findings derived from systematic reviews and meta-analyses, offering a consolidated overview of the current state of radiomics across major clinical domains, including diagnosis, prognosis, radiogenomics, and treatment response. Building on this foundation, the second section (Section 4.2) extracts and systematizes emerging methodological and translational recommendations (R1–R6), which represent the central analytical backbone of the study and summarize the main recurring limitations and needs identified in the literature.

In Section 4.3, these recommendations are operationalized as an interpretative framework to guide the analysis of complementary primary empirical studies, allowing a structured connection between secondary evidence and real-world clinical research. This section is further expanded to explicitly include the role of methodological, standardization, and regulatory frameworks, recognizing that clinical translation depends not only on empirical performance but also on enabling infrastructures, governance systems, and policy alignment.

Section 4.4 then provides the methodological justification for adopting a narrative review design, emphasizing its suitability for integrating highly heterogeneous evidence streams that include systematic reviews, primary clinical studies, AI-based modelling approaches, and international policy documents. In contrast to systematic or scoping reviews, this approach allows for higher-order synthesis and cross-domain interpretation, which are essential in a rapidly evolving and interdisciplinary field such as radiomics.

The discussion then moves to Section 4.5, which explores future perspectives, clinical implications, and translational challenges. This section situates the current evidence within the broader trajectory of lung cancer management, highlighting both the potential of radiomics and AI to transform clinical pathways and the persistent barriers that limit their routine implementation, including standardization gaps, validation limitations, and regulatory complexity.

Finally, Section 4.6 critically addresses the limitations of the study, including the predominance of secondary evidence sources, the interpretative nature of the narrative synthesis, and the inclusion of heterogeneous policy and regulatory documents. Together, these limitations are framed not as weaknesses of the conceptual approach, but as intrinsic constraints of a rapidly evolving field in which evidence is still in the process of maturation and consolidation.

### 4.1. Key Findings Across Systematic Evidence

Given the rapidly evolving and heterogeneous nature of radiomics research in lung cancer, a narrative review of systematic reviews and meta-analyses was considered the most appropriate approach. Unlike conventional systematic reviews focused on narrowly defined questions, this methodology enables a higher-level integration of evidence across multiple application domains. It allows the identification of cross-cutting themes, shared methodological limitations, and emerging translational trends within a fragmented and rapidly expanding field [48,49,50].

This narrative synthesis of systematic reviews provides a comprehensive and integrated overview of the current landscape of radiomics in thoracic imaging for lung cancer. Across the included evidence, radiomics consistently emerges as a promising quantitative approach capable of extracting clinically relevant information beyond conventional visual image interpretation, with applications spanning diagnosis, molecular characterization, prognostic stratification, and treatment response assessment.

*From a diagnostic perspective*, several systematic reviews highlighted the high accuracy of radiomics models in differentiating benign from malignant pulmonary lesions and in supporting histological classification, particularly when combined with advanced imaging techniques or machine learning approaches [22,24,41]. Radiomics also demonstrated potential in identifying specific pathological features, such as tumor spread patterns and aggressiveness, although variability across studies limits comparability [28,31,36].

*A major area of application concerns radiogenomics*, where radiomics is used to predict molecular and genetic alterations, including EGFR, ALK, KRAS mutations, PD-L1 expression, and proliferation indices such as Ki-67. Across multiple systematic reviews and meta-analyses, these models showed promising predictive performance, often enhanced by AI techniques [21,27,30,32,38,39,42]. However, the strength of this evidence is tempered by methodological heterogeneity and limited external validation.

Radiomics also demonstrated significant potential in *prognostic stratification*, particularly in patients with advanced disease and those undergoing immunotherapy or targeted treatments. Several reviews reported associations between radiomic features and survival outcomes, including overall survival and progression-free survival [29,40,43,44]. Emerging approaches such as longitudinal and delta radiomics further suggest added value in capturing temporal changes and improving predictive accuracy [40]. Nonetheless, most of the available evidence is based on retrospective analyses, limiting generalizability.

In the context of *treatment response and monitoring*, radiomics-based models showed encouraging results in predicting response to chemoimmunotherapy and in supporting radiotherapy assessment, including the use of imaging acquired during treatment [23,25]. These findings reinforce the potential role of radiomics as a non-invasive tool for dynamic disease evaluation.

Despite these promising applications, a consistent finding across the included systematic reviews is substantial methodological variability and limited reproducibility, both of which constrain the clinical applicability of current findings. Issues such as heterogeneity in imaging protocols, variability in feature extraction, and lack of standardized validation approaches are recurrently reported [34,35,36,45,46].

The added value of the present narrative review lies in its cross-cutting synthesis of systematic evidence, which enables the identification of recurring themes, shared limitations, and converging trends across different application domains. By integrating findings from multiple systematic reviews and meta-analyses, this work moves beyond single-study interpretations and provides a more structured understanding of the current state of radiomics in lung cancer imaging.

Overall, while radiomics demonstrates considerable potential across multiple clinical domains, the evidence collectively suggests that the field is still in a transitional phase, moving from exploratory research toward more robust, standardized, and clinically oriented applications.

### 4.2. Emerging Recommendations

Synthesized evidence from the included systematic reviews indicates a methodologically active and rapidly evolving field, albeit one constrained by structural limitations that compromise both reproducibility and clinical translation. These limitations appear consistently across diagnostic, prognostic, radio-genomic, and treatment-response applications, reflecting systemic rather than isolated underlying issues.

A primary and recurrent challenge is methodological heterogeneity. Substantial variability is reported across imaging acquisition protocols, reconstruction parameters, segmentation strategies, and radiomics feature extraction workflows, particularly in CT-, DECT-, and CBCT-based studies [22,25,28,38,42]. This heterogeneity limits comparability across studies and reduces the possibility of establishing robust, generalizable evidence (R1).

A second major limitation is the lack of external validation. Many models are developed and tested within retrospective, single-center datasets, with only a minority of studies including independent or multicentric validation cohorts [23,30,35,39]. This restricts the generalizability of findings and contributes to performance instability when models are applied to external populations (R2).

Closely related to this is the issue of methodological quality and risk of bias. Several reviews highlight suboptimal study design, incomplete reporting, and variability in analytical strategies, which collectively reduce the reliability of reported performance metrics [34,35,36]. These limitations are particularly evident in early-phase radiomics investigations where standardized protocols are not consistently applied (R3).

From a modelling perspective, the limited generalizability of AI-based approaches represents a critical barrier. Although machine learning and deep learning models frequently achieve strong internal performance, their accuracy often decreases in external validation settings due to dataset shift and population variability [30,39]. This indicates a dependency on local data characteristics and underscores the need for more robust training frameworks (R4).

Despite these limitations, the included evidence consistently highlights that performance improvements are frequently achieved when radiomics is combined with clinical or genomic information, supporting the value of multimodal integration strategies (R5).

In addition, the limited interpretability of AI models remains a relevant challenge. Deep learning approaches, while performant, often operate as non-transparent systems, limiting their acceptance in clinical environments where interpretability and explainability are essential for decision-making (R6).

As summarized in Table 2, these observations converge into a set of emerging recommendations for future development and clinical translation.

### 4.3. Clinical Translation Pathways in Radiomics and AI for Lung Cancer Imaging

This section complements the overall synthesis of the review by extending the discussion toward the translational dimension of radiomics and AI in lung cancer imaging, moving beyond methodological evidence toward clinical implementation pathways.

Despite substantial advances in radiomics and AI-based approaches for lung cancer imaging, the translational pathway toward routine clinical implementation remains far from complete. As highlighted by the broader clinical context of lung cancer imaging and early detection [2,3,4,5], a persistent gap remains between methodological innovation and real-world clinical adoption. Within this framework, clinical translation should be understood as a multi-layered and iterative process rather than a linear progression, in which different evidence-generating and enabling components contribute in a complementary and synergistic way.

In particular, the translational pathway can be conceptually framed around two interconnected domains. The first includes *primary clinical studies*, predominantly based on CT and radiotherapy imaging, which represent the main source of methodological development, hypothesis generation, and early validation of radiomics and AI models. The second domain concerns *policy and standardization frameworks*, which provide the structural, technical, and regulatory conditions necessary to ensure reproducibility, interoperability, and eventual clinical implementation of these technologies [18,19]. Although functionally distinct, these two domains are tightly interdependent, as methodological advances require standardized environments to become clinically transferable, while regulatory and policy frameworks depend on robust clinical evidence to be effectively defined and implemented.

The following sections therefore separately explore these two complementary dimensions: first, the role of primary clinical studies as the foundational evidence base for radiomics and AI in lung cancer imaging, and second, the contribution of policy and standardization initiatives in enabling their safe, reproducible, and clinically meaningful translation.

#### 4.3.1. Empirical Evidence Supporting Translational Recommendations in Lung Cancer Radiomics

Within the translational framework of radiomics and AI in lung cancer imaging, the studies included in this synthesis [51,52,53,54,55,56,57,58,59,60,61,62,63,64,65,66,67,68,69,70] were selected using the same composite keyword strategy previously adopted for the selection of the systematic reviews and metanalysis. This ensured methodological consistency across the different levels of evidence synthesis and alignment with the overarching structure of the overview core. Taken together, these studies provide a coherent and structured body of evidence spanning the main stages of the radiomics workflow, including lesion detection, tumor characterization, prognostic modeling, treatment response prediction, and radiomics–omics integration.

A first group of studies focuses on early lesion characterization and diagnostic stratification, particularly the differentiation between benign and malignant pulmonary lesions and the assessment of indeterminate nodules. CT- and diffusion-based radiomics approaches demonstrate consistent utility in improving diagnostic discrimination and supporting clinical decision-making in early-stage evaluation [53,68,70]. These contributions are mainly associated with R1 (standardization of imaging protocols/workflows) and R3 (methodological rigor and bias reduction), emphasizing the importance of acquisition harmonization, segmentation reproducibility, and feature robustness.

A second thematic area concerns tumor characterization and pathological inference, where radiomics is used to predict histological subtype, tumor invasiveness, and biological aggressiveness. PET/CT- and CT-based machine learning models increasingly demonstrate the ability to associate imaging-derived features with clinically relevant pathological endpoints [54,58,64]. These studies primarily support R4 (robust and generalizable AI models), highlighting the need for reproducible analytical pipelines and validation across heterogeneous clinical settings.

A third area of evidence relates to prognostic modeling and survival prediction, including overall survival, progression-free survival, and metastatic risk stratification. Radiomic signatures show consistent potential in stratifying patients across different disease stages, including locally advanced and oligometastatic non-small cell lung cancer [51,59,66]. This body of evidence mainly contributes to R2 (external and multicentric validation) and R4, reinforcing the importance of independent validation and clinical generalizability.

In parallel, several studies address treatment response prediction and biologically informed prognostic assessment, particularly in immunotherapy, chemoradiotherapy, and targeted therapy settings. Radiomics-based models and nomograms integrate imaging biomarkers with clinical variables to identify responders, predict tumor biology, and support personalized clinical decision-making [56,61,67,69]. These findings are directly aligned with R5 (multimodal data integration: radiomics + clinical + genomic data), reflecting the ongoing transition toward integrated predictive frameworks.

Finally, a subset of studies explores radiomics–omics integration and translational modeling, including correlations between imaging features and liquid biopsy genomic data, as well as hybrid clinical-biological-radiomics approaches [52,55]. These contributions are particularly relevant to R5 and R6 (model interpretability and transparency), emphasizing the importance of biologically grounded and explainable AI systems for clinical translation.

Overall, the analyzed literature supports a progressive transition from isolated radiomics applications toward a structured translational ecosystem in lung cancer imaging. Each study contributes in a complementary manner to the methodological and clinical recommendations summarized in Table 3, collectively supporting the evolution from methodological development to clinically actionable AI-driven tools. Several studies show partial overlap across different translational recommendations, reflecting the intrinsically multidimensional nature of radiomics applications.

The mapping between included studies and the translational recommendations (R1–R6) is summarized in Table 3.

#### 4.3.2. Methodological, Standardization, and Regulatory Frameworks Underpinning Translational Recommendations in Lung Cancer Radiomics

Beyond primary radiomics studies, a second critical polarity concerns the gap between methodological innovation and the availability of enabling regulatory, standardization, and governance frameworks. Within this landscape, a first group of initiatives is directly embedded in the radiomics and quantitative imaging domain and is explicitly designed to support feature reproducibility, harmonization, and model validation. In particular, the Image Biomarker Standardisation Initiative (IBSI) [71] provides formal definitions and computational standards for radiomic feature extraction, while the Quantitative Imaging Biomarkers Alliance (QIBA) [72] develops structured protocols for imaging acquisition, reconstruction, and biomarker validation across clinical studies. In the same line, reporting guidelines such as TRIPOD [73]—although not radiomics-specific per se—are widely applied to radiomics prediction models, ensuring transparent reporting and enabling critical appraisal and external validation of AI-driven imaging studies.

A second group of documents operates at the level of clinical translation and system integration, rather than being radiomics-specific, but has direct implications for radiomics-based AI tools. Professional societies in nuclear medicine and imaging, including the European Association of Nuclear Medicine (EANM) and the Society of Nuclear Medicine and Molecular Imaging (SNMMI) [74], issue domain-specific recommendations that influence how quantitative imaging biomarkers are interpreted, validated, and integrated into clinical workflows.

Finally, a third layer is represented by broader regulatory and governance frameworks for AI in healthcare, which indirectly but substantially shape the deployment of radiomics-based systems. The U.S. Food and Drug Administration (FDA) regulatory framework for Software as a Medical Device (SaMD) and AI/ML-based software defines pathways for clinical validation and approval of digital health technologies [75], while the European Union Artificial Intelligence Act establishes a risk-based framework for AI systems in medical contexts [76]. In parallel, World Health Organization guidance on AI in health emphasizes ethical principles, transparency, and equity in deployment, providing a global normative reference for AI-driven clinical technologies [77]. Collectively, these layered frameworks delineate a multi-level governance structure that supports radiomics translation, while still revealing persistent gaps between technical standardization, regulatory readiness, and clinical implementation.

Collectively, these layered frameworks delineate a multi-level governance structure that supports radiomics translation, while still revealing persistent gaps between technical standardization, regulatory readiness, and clinical implementation.

In order to synthesize how these different methodological, clinical, and regulatory dimensions contribute to overcoming the translational barriers identified in lung cancer radiomics, the evidence discussed above is mapped against the main methodological and implementation-oriented recommendations (R1–R6) summarized in Table 4. This mapping highlights how standardization initiatives primarily address issues of reproducibility and methodological rigor (R1–R3), while validation frameworks and multicentric efforts contribute to robustness and generalizability (R4). In parallel, integrative approaches linking imaging, clinical, and multi-omics data support multimodal integration strategies (R5), and emerging governance and regulatory instruments increasingly emphasize transparency, interpretability, and safe clinical deployment (R6).

Table 4 provides a structured overview of this alignment between evidence domains and translational recommendations.

### 4.4. Rationale for the Narrative Review Design and Integrative Synthesis Approach

The selection of a narrative review design was grounded in the intrinsic heterogeneity, interdisciplinarity, and rapid methodological evolution of radiomics research in lung cancer imaging. The available evidence spans a wide range of study types, including diagnostic, prognostic, radiogenomic, and treatment-response studies, as well as AI-driven methodological developments, together with international policy and standardization documents. This results in a fragmented and highly heterogeneous body of literature, characterized by variability in imaging protocols, feature extraction workflows, modelling strategies, and clinical endpoints.

Systematic reviews are primarily designed to address narrowly defined questions under conditions of methodological homogeneity and are therefore limited in their ability to capture cross-domain translational patterns in rapidly evolving fields such as radiomics. Scoping reviews, while useful for mapping the breadth of available literature, remain predominantly descriptive and do not support higher-order synthesis across methodological, clinical, and regulatory dimensions.

In contrast, the narrative review approach [48,49,50] enables a flexible and interpretative synthesis that is particularly suited to heterogeneous and fast-moving domains. In radiomics, where technological innovation, clinical validation, and methodological standardization evolve in parallel but not synchronously, a more integrative approach is required to capture the full complexity of the field.

A distinctive feature of the present study lies in its multilevel integrative synthesis strategy. Rather than limiting the analysis to descriptive aggregation of findings, evidence from systematic reviews and meta-analyses was first used to extract emerging methodological and translational recommendations, structured into transversal domains (R1–R6). These recommendations were then used as an interpretative framework to guide the analysis of primary studies, including clinical and observational research, enabling a structured evaluation of how methodological needs translate into empirical evidence.

In addition, international and institutional policy documents and standardization frameworks were incorporated to contextualize the translational feasibility of radiomics-based AI systems. This allowed for a third analytical layer, linking empirical evidence with governance, interoperability, and implementation constraints, which are critical for clinical translation but are not captured by traditional evidence synthesis designs.

This sequential and bidirectional synthesis strategy enabled an iterative integration process, where secondary evidence informed the interpretation of primary studies, while empirical and policy-level evidence contributed to refining and contextualizing overarching methodological recommendations. This design supports a more comprehensive and system-level understanding of radiomics in lung cancer imaging, bridging clinical applications, AI methodologies, and translational governance.

Overall, the narrative design was not adopted as a substitute for systematic synthesis, but as a deliberate methodological choice to enable cross-level integration across heterogeneous evidence streams. This approach allows the identification of converging translational barriers and enabling factors that would not emerge from systematic or scoping methodologies alone, thereby providing a more complete and operationally relevant understanding of radiomics-based AI in lung cancer imaging.

### 4.5. Future Perspectives, Clinical Implications and Translational Challenges

The field of lung cancer is currently undergoing a progressive but profound transformation, driven by advances in early detection strategies, imaging technologies, artificial intelligence (AI), and radiomics. Despite these rapid developments, their effective integration into routine clinical practice remains incomplete, and several methodological, organisational, and translational challenges continue to limit full implementation.

#### 4.5.1. Towards Earlier and More Precise Lung Cancer Detection

Lung cancer remains the leading cause of cancer-related mortality worldwide, largely due to the persistence of late-stage diagnosis in a significant proportion of patients [1]. Evidence from large-scale screening trials has demonstrated that CT screening and LDCT can significantly reduce lung cancer mortality in high-risk populations [5,78], supporting its role as a cornerstone of early detection strategies.

However, the implementation of LDCT screening in real-world settings remains heterogeneous. Principal limitations include a high rate of false positives, sub- optimal population stratification, and organizational and resource constraints that restrict scalability.

In parallel, biomarker-based approaches are being actively investigated as complementary tools to improve early detection accuracy and patient selection, although their clinical validation is still ongoing and not yet fully established [2].

#### 4.5.2. Standardisation and Methodological Considerations

A major limitation in radiomics research remains the lack of full methodological standardisation. In response to this issue, international initiatives such as the Image Biomarker Standardisation Initiative (IBSI) and the Quantitative Imaging Biomarkers Alliance (QIBA) have provided essential frameworks to harmonise feature definitions and improve imaging biomarker reliability [18,72]. Similarly, reporting guidelines such as TRIPOD aim to enhance transparency, reproducibility, and methodological rigor in predictive modelling studies [73].

Despite these advances, variability in CT acquisition parameters, reconstruction settings, and segmentation approaches continues to significantly affect radiomic feature stability [14,15,17]. This underscores the need for further methodological refinement, particularly in the following domains:

- Harmonisation of imaging protocols across centres;
- Robust and reproducible feature selection strategies;
- Rigorous external and multicentric validation;
- Explicit quantification of uncertainty, bias, and model drift.

Only through systematic standardisation efforts will it be possible to ensure clinical reliability and comparability across studies.

#### 4.5.3. Clinical Implications and Integration into Practice

From a clinical perspective, radiomics- and AI-based tools are progressively positioning themselves as potential decision-support systems across the entire lung cancer care pathway, including screening, diagnosis, staging, and treatment planning. Promising applications include the prediction of lymph node involvement, tumour aggressiveness, molecular alterations, and response to systemic therapies [11,12].

However, despite these encouraging developments, clinical adoption remains limited. This gap is primarily related to the absence of large-scale prospective validation studies, variability in model performance across institutions, and difficulties in integrating AI tools into existing clinical workflows in a transparent and interpretable manner. As a result, most radiomics applications currently remain in the investigational or pre-implementation phase, albeit with increasing proximity to clinical translation.

#### 4.5.4. Regulatory and Ethical Considerations

The implementation of AI-based systems in healthcare is increasingly shaped by evolving regulatory and ethical frameworks. The European Artificial Intelligence Act establishes requirements related to transparency, safety, and accountability for high-risk AI systems in healthcare contexts [76]. In parallel, the U.S. Food and Drug Administration provides regulatory guidance for Software as a Medical Device (SaMD), defining pathways for clinical validation and approval [75].

In addition, the World Health Organization has emphasised the importance of responsible AI governance in healthcare, with particular attention to equity, data protection, and patient safety [77]. These considerations are becoming increasingly relevant as AI-driven tools transition from research settings to real-world clinical environments.

#### 4.5.5. Concluding Perspective

Overall, lung cancer management is progressively evolving towards a more integrated and data-driven paradigm, in which imaging, clinical variables, and molecular information are combined through advanced computational approaches. While technological progress in this field is substantial, the principal challenge is no longer model development alone, but rather robust, reproducible, and clinically meaningful translation.

Future progress will therefore depend on the convergence of methodological standardisation, multicentric validation, interdisciplinary collaboration, and regulatory alignment. Only through this integrated effort will it be possible to ensure that AI and radiomics-based innovations can effectively and safely support clinical decision-making in lung cancer care.

### 4.6. Limitations

Despite the comprehensive and integrative design adopted in this work, several limitations should be acknowledged. First, the evidence base is primarily derived from secondary structured sources, specifically systematic reviews and meta-analyses. While this approach allows for a high-level synthesis of consolidated findings, it inherently depends on the methodological quality, reporting standards, and heterogeneity of the included secondary studies. As a result, any limitations present at the level of primary investigations are indirectly propagated into the present synthesis.

Second, the narrative review approach, while essential and methodologically justified for addressing the heterogeneity and interdisciplinary nature of the field, is inherently characterized by broader interpretative “looser” boundaries compared to systematic or scoping reviews. This flexibility represents a key strength in terms of conceptual integration and cross-domain synthesis, but it also introduces a degree of subjectivity in evidence interpretation and limits reproducibility in a strictly protocol-driven framework.

Third, although this narrative review incorporates an additional layer of interpretation through the use of emerging recommendations (R1–R6) and complementary primary studies selected with a consistent methodological framework, this integration remains interpretative rather than quantitative. Consequently, future developments in the field, alongside the progressive expansion of available medical knowledge, are expected to enable more focused and domain-specific analyses, supported by a growing number of high-quality prospective and multicentric studies that will strengthen evidence robustness and comparability.

Fourth, the synthesis includes evidence derived from peer-reviewed publications indexed in international scientific databases as well as relevant policy, regulatory, and standardization documents issued by international organizations and scientific societies. While this ensures coverage of both empirical and governance dimensions of the field, it may still be influenced by differences in regional regulatory frameworks, reporting practices, and accessibility of documents. In addition, some emerging international initiatives may not yet be fully represented due to their evolving or non-indexed status. Future updates of this work may benefit from broader and continuously updated inclusion of both scientific and policy-level sources.

## 5. Conclusions

Lung cancer imaging is currently undergoing a major transformation driven by AI, radiomics, and quantitative imaging, which are reshaping thoracic oncology. The evidence synthesized in this review shows that radiomics has moved beyond an exploratory phase and is now a maturing methodological framework with clear potential clinical value across multiple stages of lung cancer management. Across the included systematic reviews, radiomics consistently emerges as a promising quantitative approach capable of extracting clinically relevant information beyond conventional imaging interpretation, with applications spanning diagnosis, radiogenomics, prognostic stratification, and treatment response assessment. However, the evidence is simultaneously characterized by substantial methodological heterogeneity, limited reproducibility, and restricted external validation, which collectively constrain clinical transferability.

However, translation into routine clinical practice remains incomplete. Although radiomics models show strong performance in diagnostic, prognostic, and radiogenomic tasks, important limitations persist, particularly regarding reproducibility, external validity, and standardization. These challenges are systemic and largely reflect variability in imaging protocols, biological tumour heterogeneity, and heterogeneous analytical pipelines.

In conclusion, lung cancer radiomics and AI are positioned at a critical transition point between innovation and implementation. Their full clinical impact will depend on coordinated efforts in standardisation, multicentric validation, interdisciplinary collaboration, and regulatory integration, with a shift toward robust, generalisable, and clinically embedded systems.

## Figures and Tables

**Table 1:** Overview of included systematic reviews and main thematic areas.

Study	Contribution of Radiomics	Field of Interest
Fuster-Matanzo et al. [21]	Radiomics for oncogenic mutation prediction (EGFR, ALK, KRAS)	Radiogenomics
Zhang J. et al. [22]	DECT radiomics for diagnosis and invasiveness	Diagnosis
Chen H. et al. [23]	Radiomics predicting response to chemoimmunotherapy	Treatment response
Nayak et al. [24]	Histological subtype classification	Diagnosis
Chang et al. [25]	CBCT radiomics for radiotherapy monitoring	Treatment monitoring
Sahrai et al. [26]	Differentiating cancer from tuberculosis	Diagnosis
Salimi et al. [27]	Radiomics for PD-L1 prediction	Radiogenomics
Chen et al. [28]	STAS prediction	Diagnosis
Zhang Y.R. et al. [29]	Prognostic stratification in immunotherapy	Prognosis
Liu et al. [30]	ML/DL for EGFR prediction	Radiogenomics
Chen J. et al. [31]	STAS detection	Diagnosis
Shahidi et al. [32]	Ki-67 prediction	Radiogenomics
Jiang et al. [33]	Radiogenomics for prognosis	Prognosis
Tran et al. [34]	Methodological quality assessment	Methodology
Jia et al. [35]	Prognostic models quality assessment	Prognosis
Cheng et al. [36]	Prediction of aggressiveness features	Diagnosis
Chen J. et al. [37]	Mutation prediction models	Radiogenomics
Luo et al. [38]	Ki-67 prediction	Radiogenomics
Nguyen et al. [39]	AI-based EGFR prediction	Radiogenomics
Chiu et al. [40]	Delta radiomics for prognosis	Prognosis
Shi et al. [41]	Malignancy prediction in nodules	Diagnosis
Felfli et al. [42]	EGFR prediction	Radiogenomics
Wang T.W. et al. [43]	Prognosis with targeted therapy	Prognosis
Wu et al. [44]	Peritumoral radiomics for prognosis	Prognosis
Lee et al. [45]	Recurrence prediction after SABR	Prognosis
Zhang et al. [46]	Immunotherapy biomarkers	Prognosis
van Laar et al. [47]	CT prognostic factors	Prognosis

**Table 2:** Emerging Recommendations Derived from Systematic Evidence Synthesis.

Code	Emerging Recommendation	Evidence Basis
R1	Standardization of imaging protocols and radiomics workflows	Variability in CT, DECT, and CBCT acquisition and feature extraction [22,25,28,38,42]
R2	Strengthening of external and multicentric validation	Predominance of retrospective single-centre studies and limited validation cohorts [23,30,35,39]
R3	Improvement of methodological rigor and reduction in bias	Suboptimal study design, incomplete reporting, variability in analytical strategies [34,35,36]
R4	Development of more robust and generalizable AI models	Domain shift and reduced performance in external validation [30,39]
R5	Integration of multimodal data (radiomics + clinical + genomic)	Improved predictive performance with combined approaches [21,30,33]
R6	Enhancement of model interpretability and transparency	Black-box limitations of deep learning models [30,39]

**Table 3:** Mapping of included studies [51,52,53,54,55,56,57,58,59,60,61,62,63,64,65,66,67,68,69,70] to translational recommendations (R1–R6).

Translational Recommendation	Thematic Focus	Included Studies	How They Contribute
R1	Image acquisition consistency, feature robustness, reproducibility	[53,54,57,70]	Address variability in CT/PET/diffusion imaging, emphasize robustness of radiomic features and standardized pipelines for reliable model development
R2	Generalizability and independent cohort testing	[51,54,59,63,66]	Provide validation of radiomic models across multicenter cohorts or clinical trials, supporting robustness and transferability of predictive performance
R3	Study design, feature stability, reduction of overfitting	[53,57,68,70]	Focus on robust feature selection, variability assessment, and methodological control to reduce bias and improve reproducibility
R4	Diagnostic, pathological characterization, and prognostic performance	[54,58,59,60,64,65,66,67,69]	Develop and validate machine learning and radiomics models for diagnosis, pathological characterization, prognostic prediction, and clinically relevant outcome assessment across NSCLC and related lung cancer phenotypes.
R5	Integrated predictive modeling	[52,55,56,61,67,69]	Combine radiomics with clinical, biological, genomic, or immunological data to improve the prediction of response, prognosis, and tumor behavior
R6	Explainability and clinical interpretability	[55,56,59,61]	Promote interpretable radiomic signatures and clinically explainable models, including radiomics scores and clinico-biological frameworks

**Table 4:** Mapping of standardization, clinical, and governance frameworks to translational recommendations (R1–R6) in lung cancer radiomics.

Framework/Initiative	Type	Main Focus	Contribution to Radiomics Translation	Addressed Recommendations
IBSI (Image Biomarker Standardisation Initiative) [71]	Radiomics standardization	Radiomic feature definition and extraction harmonization	Ensures reproducible feature computation and reduces methodological variability across studies	R1, R3
QIBA (Quantitative Imaging Biomarkers Alliance) [72]	Imaging standardization	Imaging acquisition, reconstruction, and biomarker validation protocols	Improves cross-institutional consistency and supports robust quantitative imaging workflows	R1, R4
TRIPOD [73]	Reporting guideline	Transparent reporting of predictive models	Enhances reproducibility, external validation, and methodological transparency of radiomics models	R2, R3
EANM/SNMMI guidelines [74]	Clinical society guidance	Nuclear medicine imaging and quantitative biomarker use	Supports clinical interpretation and integration of imaging biomarkers into practice	R4, R6
FDA (SaMD) [75]	Regulatory framework	Clinical validation and approval pathways for AI/software	Defines regulatory requirements for clinical deployment of AI-based medical software, including radiomics applications	R6
EU AI Act [76]	AI regulation	Risk-based regulation of AI systems in healthcare	Ensures safety, transparency, accountability, and risk stratification for clinical AI systems	R6
WHO AI governance principles [77]	Global governance	Ethical, equitable, and transparent AI use in health	Provides an overarching ethical framework for the responsible deployment of AI-driven radiomics	R6

## Data Availability

No new data were created or analyzed in this study. Data sharing is not applicable to this article.

## Supplementary Materials

The following supporting information can be downloaded at https://www.mdpi.com/article/10.3390/jimaging12070287/s1: Section S1: Complementation of the analysis; Table S1: Contribution of Radiomics, Role of AI, and Opportunities.
