# Supplementary material for: Radiomics in Lung Cancer Imaging: A Narrative Review of Current Evidence
Source: J Imaging. 2026 Jun 29;12(7):287. doi: 10.3390/jimaging12070287 (PMC13412686; doi:10.3390/jimaging12070287)
Supplement: Supplementary file 1 [file jimaging-12-00287-s001.zip › jimaging-4329661-supplementary.pdf]

Journal of Imaging

MDPI

Review

Radiomics in lung cancer imaging: A narrative review of current evidence

Andrea Lastrucci [1], Nicola Iosca[1] , Edoardo Cavigli[2], Diletta Cozzi[2], Angelo Barra[1], Yannick Wandael[1], Cosimo Nardi[3], Renzo Ricci[1], Vittorio Miele[2,3] and Daniele Giansanti [4,*]

[1] Department of Allied Health Professions, Azienda Ospedaliero-Universitaria Careggi, 50134 Florence, Italy (ioscan@aou-careggi.toscana.it, wandaely@aou-careggi.toscana.it, barraa@aou-careggi.toscana.it,, riccire@aou-careggi.toscana.it)
[2] Department of Emergency Radiology, Careggi University Hospital, L.Go Brambilla 3, 50123, Florence, Italy (caviglie@aou-careggi.toscana.it, cozzid@aou-careggi.toscana.it)
[3] Department of Experimental and Clinical Biomedical Sciences "Mario Serio", University of Florence, Florence, Italy (Cosimo.nardi@unifi.it, vmiele@sirm.org)
[4] Centre IATIS, Istituto Superiore di Sanità, 00161 Rome, Italy (daniele.giansanti@iss.it)
* Correspondence: daniele.giansanti@iss.it ;

Academic Editor: Stephen Pistorius

Received: 5 May 2026
Revised: 15 June 2026
Accepted: 19 June 2026
Published: 29 June 2026

Copyright: © 2026 by the authors. Licensee MDPI, Basel, Switzerland. This article is an open access article distributed under the terms and conditions of the Creative Commons Attribution (CC BY) license.

Section S1. Complementation of the analysis

Fuster-Matanzo et al. [21] analyzed studies involving NSCLC patients undergoing pre-treatment CT-based radiomics and AI modelling to predict oncogenic mutations (EGFR, ALK, KRAS). The primary outcomes were sensitivity and false positive rate. The review highlighted good predictive performance of radiomics models, with only marginal improvement from integrating clinical data. However, limitations included heterogeneity in model development, lack of standardization, and absence of significant predictors in meta-regression analyses.

Zhang J. et al. [22] evaluated studies for the assessment of lung lesions using dual-energy CT (DECT), either alone or combined with radiomics. Outcomes included sensitivity, specificity, and AUC for malignancy and invasiveness. The review highlighted high diagnostic accuracy, particularly with integrated radiomics models. Key limitations included heterogeneity in feature extraction methods and radiomics algorithms, which were major sources of variability.

- Chen H. et al. [23] assessed studies involving NSCLC patients treated with neoadjuvant chemoimmunotherapy, using preoperative CT-based radiomics models to predict pathological response. Outcomes included AUC, sensitivity, and specificity. The review highlighted good predictive performance, especially for deep learning models. Limitations included variability in study quality, differences in feature selection methods, and reduced performance in external validation cohorts.
- Nayak et al. [24] reviewed studies assessing pre-treatment histological subtype classification with CT-based radiomics and machine learning or deep learning models in NSCLC patients. The primary outcome was diagnostic accuracy. The review highlighted strong classification performance, particularly with deep learning methods. Limitations included small sample sizes, heterogeneity across studies, and limited external validation.
- Chang et al. [25] included and analyzed studies using radiomics on CBCT images during treatment or follow-up to evaluate tumor response, toxicity, and survival in cancer patients undergoing image-guided radiotherapy. The review highlighted the potential of CBCT-based radiomics for treatment monitoring. However, limitations included image quality issues, variability in acquisition protocols, and lack of standardization affecting reproducibility.

- Sahrai et al. [26] evaluated studies in which CT-based radiomics models and combined radiomics + clinical models were used to differentiate lung cancer from tuberculosis among patients with pulmonary lesions. Outcomes included sensitivity, specificity, and AUC. The review highlighted superior performance of combined models. Limitations included heterogeneity due to CT acquisition parameters, reduced performance in external validation, and retrospective study designs.
- Salimi et al. [27] analyzed studies including NSCLC patients undergoing pretreatment CT-based radiomics for PD-L1 expression prediction. Outcomes included AUC, sensitivity, and specificity. The review highlighted promising predictive ability. However, limitations included high risk of bias (QUADAS-2), methodological inconsistencies, and suboptimal study design quality.
- Chen et al. [28] investigated studies in which lung cancer patients were assessed with preoperative CT-based radiomics for predicting STAS. Outcomes included pooled sensitivity, specificity, and AUC. The review highlighted moderate diagnostic accuracy. Limitations included moderate methodological quality, heterogeneity, and lack of large prospective multicenter validation.
- Zhang Y.R. et al. [29] analyzed studies in which pretreatment CT texture analysis was used to evaluate prognostic stratification in advanced NSCLC patients undergoing immunotherapy. Outcomes included OS and PFS. The review highlighted significant prognostic stratification. Limitations included retrospective design, variability in radiomic features, and heterogeneity in included studies.
- Liu et al. [30] assessed recent ML and DL approaches for predicting EGFR mutation status in NSCLC, reporting higher internal performance for deep learning models (AUC 0.84) compared to radiomics-based methods. However, the study highlighted key limitations, including reliance on single-centre datasets, limited external validation (only 29% of studies), and performance drops due to domain shift. Additional barriers such as high risk of bias, lack of standardized protocols, and poor model transparency restrict current clinical applicability.
- Chen J. et al. [31] analyzed studies including lung cancer patients assessed with CT-based radiomics models for STAS detection. Outcomes included sensitivity, specificity, and AUC. The review highlighted consistent diagnostic performance. Limitations included high heterogeneity and variability between development and validation cohorts.
- Shahidi et al. [32] examined studies that assessed pre-treatment Ki-67 prediction by CT-based radiomics analysis in NSCLC patients. Outcomes included pooled sensitivity and specificity. The review highlighted promising predictive accuracy. Limitations included heterogeneity in segmentation tools and study design differences.
- Jiang et al. [33] analyzed studies assessing lung cancer patients with radiogenomics models (CT radiomics + genomic data) for prognosis prediction. Outcomes included AUC and C-index. The review highlighted improved performance of combined models. Limitations included small sample sizes, retrospective designs, and a lack of standardized datasets.
- Tran et al. [34] conducted a systematic review of radiomics studies in post-radiotherapy stage III/IV NSCLC, aiming to identify imaging biomarkers and assess methodological quality using the CLEAR and RQS frameworks, as well as a combined CLEAR–RQS tool. The review highlighted substantial heterogeneity, lack of reproducibility, and generally low research quality, with no consistent radiomic biomarkers identified. Key limitations included low RQS scores, limited validation, insufficient data and code sharing, and a lack of standardized protocols.
- Jia et al. [35] examined studies that evaluated prognosis prediction using CT-based radiomics in NSCLC patients. Outcomes included survival metrics and RQS

https://doi.org/10.3390/jimaging12070287

assessment. The review highlighted low methodological quality and limited validation. Limitations included low RQS scores, retrospective designs, and insufficient external validation.

- Cheng et al. [36] investigated studies in which lung cancer patients were assessed with a preoperative CT radiomic model for predicting histopathological aggressiveness features. Outcomes included diagnostic accuracy. The review highlighted inconclusive evidence. Limitations included high risk of bias, lack of external validation, and poor methodological quality.
- Chen J. et al. [37] analyzed studies assessing mutation prediction with radiomics-based machine learning models in NSCLC patients. Outcomes included c-index and accuracy. The review highlighted good predictive performance. Limitations included heterogeneity across imaging modalities and the influence of clinical variables.
- Luo et al. [38] analyzed CT-based radiomics models for predicting Ki-67 expression in lung cancer, demonstrating promising diagnostic performance (AUC 0.85 in training and 0.81 in validation cohorts). Several key limitations were identified, including the retrospective design of all included studies, significant heterogeneity related to imaging protocols and Ki-67 thresholds, and reduced specificity in validation datasets.
- Nguyen et al. [39] examined studies that involved EGFR mutation prediction with AI-based radiomics in NSCLC patients. Outcomes included AUC, sensitivity, and specificity. The review highlighted the superior performance of deep learning. Limitations included variability in segmentation methods and a lack of standardized AI pipelines.
- Chiu et al. [40] assessed the role of serial imaging and delta radiomics for prognostic stratification of lung cancer patients undergoing immunotherapy, demonstrating good predictive performance (AUC 0.81) and significant associations with progression-free and overall survival. However, the evidence is limited by the small number of studies, heterogeneity in imaging and feature extraction, and insufficient standardization and external validation, restricting generalizability and clinical applicability.
- Shi et al. [41] evaluated studies assessing malignancy prediction of pulmonary nodules with CT-based radiomics. Outcomes included sensitivity, specificity, and AUC. The review highlighted excellent diagnostic performance. Limitations included a high risk of bias and variability in nodule types and study design.
- Felfli et al. [42] examined studies evaluating CT-based radiomics models for EGFR mutation prediction in NSCLC patients. Outcomes included AUC and RQS. The review highlighted promising performance. Limitations included wide variability in methodological quality and lack of harmonization.
- Wang T.W. et al. [43] analyzed studies evaluating prognostic value of CT-based radiomics features in NSCLC patients treated with EGFR-TKIs. Outcomes included PFS and the concordance index. Limitations included heterogeneity in treatment protocols and retrospective study designs.
- Wu et al. [44] examined studies in which peritumoural CT radiomics features for prognosis prediction were evaluated in NSCLC patients. Outcomes included survival metrics and RQS. The review highlighted promising prognostic value. Limitations included high risk of bias, retrospective design, and lack of prospective validation.
- Lee et al. [45] evaluated radiological features for detecting local recurrence after Stereotactic Ablative Radiotherapy in NSCLC, identifying specific imaging patterns associated with recurrence. The evidence is limited by heterogeneous study designs, challenges in differentiating recurrence from radiation-induced lung injury, and scarce validation of radiomic features.
- Zhang et al. [46] assessed radiomic biomarkers for predicting immune checkpoint inhibitor outcomes, reporting moderate discriminatory performance (AUC ~0.7) but a limited evidence base. The evidence is constrained by a very small number of

https://doi.org/10.3390/jimaging12070287

heterogeneous studies, weak methodological rigor, and a lack of prospective and external validation.

- Van Laar et al. [47] evaluated CT-based prognostic factors for overall survival in stage III NSCLC, identifying tumor and nodal size-related features as the most consistent predictors. The evidence is limited by substantial heterogeneity, variable study quality, and limited validation of novel imaging biomarkers beyond established clinical factors.

To better synthesize the heterogeneous body of evidence, the included systematic reviews were categorized according to their primary application domains. This thematic organization enables the identification of recurrent patterns and emerging directions in radiomics research in lung cancer.

Table S1. Contribution of Radiomics, Role of AI, and Opportunities.

Study	*Contribution of Radiomics*	*Role of AI*	*Opportunities*
Fuster-Matanzo et al. [21]	Prediction of oncogenic mutations (EGFR, ALK, KRAS)	ML models for feature integration	Radiogenomics integration, standardization
Zhang J. et al. [22]	High diagnostic accuracy with DECT radiomics	ML improves classification	Harmonization of feature extraction
Chen H. et al. [23]	Prediction of pathological response	DL improves prediction	Prospective validation
Nayak et al. [24]	Histological subtype classification	DL enhances accuracy	Larger datasets
Chang et al. [25]	Treatment monitoring (CBCT radiomics)	AI supports longitudinal analysis	Standardized imaging protocols
Sahrai et al. [26]	Differentiating cancer from tuberculosis	ML combined models	External validation
Salimi et al. [27]	PD-L1 prediction	AI-based prediction models	Bias reduction, robust study design
Chen et al. [28]	STAS prediction	ML-based classification	Multicenter validation
Zhang Y.R. et al. [29]	Prognostic stratification (OS, PFS)	Texture-based ML models	Prospective studies
Liu et al. [30]	EGFR prediction	DL superior to radiomics	Explainable AI, generalizability
Chen J. et al. [31]	STAS detection	ML classification	Reduce heterogeneity
Shahidi et al. [32]	Ki-67 prediction	ML models	Standardized segmentation
Jiang et al. [33]	Radiogenomics prognosis	Integrated AI models	Multimodal datasets
Tran et al. [34]	Methodological assessment	Limited AI robustness	Standardization frameworks
Jia et al. [35]	Prognostic models	ML-based approaches	Improve methodological quality
Cheng et al. [36]	Aggressiveness prediction	ML classification	Reduce bias
Chen J. et al. [37]	Mutation prediction	ML models	Integration with clinical data

https://doi.org/10.3390/jimaging12070287

Luo et al. [38]	Ki-67 prediction	ML-based models	Improve specificity
Nguyen et al. [39]	EGFR prediction	DL models outperform ML	Standard AI pipelines
Chiu et al. [40]	Delta radiomics for prognosis	AI longitudinal modeling	Standardization and validation
Shi et al. [41]	Malignancy prediction	ML classification	Reduce bias
Felfli et al. [42]	EGFR prediction	ML models	Harmonization
Wang T.W. et al. [43]	Prognosis with targeted therapy	ML survival models	Prospective validation
Wu et al. [44]	Peritumoral radiomics prognosis	ML models	External validation
Lee et al. [45]	Recurrence prediction	Limited AI use	Radiomics validation
Zhang et al. [46]	Immunotherapy biomarkers	ML models	Larger datasets
van Laar et al. [47]	Prognostic CT features	Limited AI role	Integration with radiomics

Acknowledgments: The author acknowledges the use of ChatGPT (OpenAI, GPT-5-based model, free version) for English-language editing assistance during the preparation of this manuscript.

References

1. World Health Organization. Lung Cancer. Available online:https://www.who.int/news-room/fact-sheets/detail/lung-cancer (accessed on 5 May 2026).
2. Gasparri, R.; Sabalic, A.; Spaggiari, L. The Early Diagnosis of Lung Cancer: Critical Gaps in the Discovery of Biomarkers. *J. Clin. Med.* **2023**, *12*, 7244. https://doi.org/10.3390/jcm12237244. PMID: 38068296; PMCID: PMC10707119.
3. Kalinke, L.; Thakrar, R.; Janes, S.M. The promises and challenges of early non-small cell lung cancer detection: Patient perceptions, low-dose CT screening, bronchoscopy and biomarkers. *Mol. Oncol.* **2021**, *15*, 2544–2564. https://doi.org/10.1002/1878-0261.12864. PMID: 33252175; PMCID: PMC8486568.
4. Ramos, R.; Moura, C.S.; Costa, M.; Lamas, N.J.; Castro, L.P.E.; Correia, R.; Garcez, D.; Pereira, J.M.; Sousa, C.; Vale, N. Heterogeneity of Lung Cancer: The Histopathological Diversity and Tumour Classification in the Artificial Intelligence Era. *Pathobiology* **2025**, *92*, 239–250. https://doi.org/10.1159/000544892. PMID: 40222364.
5. National Lung Screening Trial Research Team; Aberle, D.R.; Adams, A.M.; Berg, C.D.; Black, W.C.; Clapp, J.D.; Fagerstrom, R.M.; Gareen, I.F.; Gatsonis, C.; Marcus, P.M.; et al. Reduced lung-cancer mortality with low-dose computed tomographic screening. *N. Engl. J. Med.* **2011**, *365*, 395–409. https://doi.org/10.1056/NEJMoa1102873. PMID: 21714641; PMCID: PMC4356534.
6. Devaraj, A.; Cook, G.J.R.; Hansell, D.M. PET/CT in non-small cell lung cancer staging—Promises and problems. *Clin. Radiol.* **2007**, *62*, 97–110.
7. Tárnoki, Á.D.; Tárnoki, D.L.; Dąbrowska, M. New developments in the imaging of lung cancer. *Breathe* **2024**, *20*, 230176. https://doi.org/10.1183/20734735.0176-2023.
8. Yoon, S.H.; Kim, Y.J.; Doh, K.; Kim, J.; Lee, K.H.; Lee, K.W.; Kim, J. Interobserver variability in Lung CT Screening Reporting and Data System categorisation in subsolid nodule-enriched lung cancer screening CTs. *Eur. Radiol.* **2021**, *31*, 7184–7191. https://doi.org/10.1007/s00330-021-07800-5. PMID: 3373368.
9. van Griethuysen, J.J.M.; Fedorov, A.; Parmar, C.; Hosny, A.; Aucoin, N.; Narayan, V.; Beets-Tan, R.G.H.; Fillion-Robin, J.C.; Pieper, S.; Aerts, H.J.W.L. Computational Radiomics System to Decode the Radiographic Phenotype. *Cancer Res.* **2017**, *77*, e104–e107. https://doi.org/10.1158/0008-5472.CAN-17-0339. PMID: 29092951; PMCID: PMC5672828.
10. Zhang, Y.; Oikonomou, A.; Wong, A.; Haider, M.A.; Khalvati, F. Radiomics-based Prognosis Analysis for Non-Small Cell Lung Cancer. *Sci. Rep.* **2017**, *7*, 46349. https://doi.org/10.1038/srep46349.

11. Wu, J.; Zhou, Y.; Xu, C.; Yang, C.; Liu, B.; Zhao, L.; Song, J.; Wang, W.; Yang, Y.; Liu, N. Effectiveness of CT radiomic features combined with clinical factors in predicting prognosis in patients with limited-stage small cell lung cancer. *BMC Cancer* **2024**, *24*, 170. https://doi.org/10.1186/s12885-024-11862-1.
12. Li, J.; Shi, Q.; Yang, Y.; Xie, J.; Xie, Q.; Ni, M.; Wang, X. Prediction of EGFR mutations in non-small cell lung cancer: A nomogram based on 18F-FDG PET and thin-section CT radiomics with machine learning. *Front. Oncol.* **2025**, *15*, 1510386. https://doi.org/10.3389/fonc.2025.1510386.
13. Rinaldi, L.; De Angelis, S.P.; Raimondi, S.; Rizzo, S.; Fanciullo, C.; Rampinelli, C.; Mariani, M.; Lascialfari, A.; Cremonesi, M.; Orecchia, R.; et al. Reproducibility of radiomic features in CT images of NSCLC patients: An integrative analysis on the impact of acquisition and reconstruction parameters. *Eur. Radiol. Exp.* **2022**, *6*, 2. https://doi.org/10.1186/s41747-021-00258-6.
14. Tunali, I.; Hall, L.O.; Napel, S.; Cherezov, D.; Guvenis, A.; Gillies, R.J.; Schabath, M.B. Stability and reproducibility of computed tomography radiomic features extracted from peritumoral regions of lung cancer lesions. *Med. Phys.* **2019**, *46*, 5075–5085. https://doi.org/10.1002/mp.13808.
15. Yang, S.X.; Li, M.; Zhou, L.N.; Hou, D.H.; Zhang, L.; Wu, N. Reproducibility of the CT radiomic features of pulmonary nodules: The effects of the CT reconstruction algorithm, radiation dose, and contrast agent. *Quant. Imaging Med. Surg.* **2025**, *15*, 2309–2318. https://doi.org/10.21037/qims-24-2026. PMID: 40160618; PMCID: PMC11948441.
16. Emaminejad, N.; Wahi-Anwar, M.W.; Kim, G.H.J.; Hsu, W.; Brown, M.; McNitt-Gray, M. Reproducibility of lung nodule radiomic features: Multivariable and univariable investigations that account for interactions between CT acquisition and reconstruction parameters. *Med. Phys.* **2021**, *48*, 2906–2919. https://doi.org/10.1002/mp.14830. PMID: 33706419; PMCID: PMC8273077.
17. Berenguer, R.; Pastor-Juan, M.D.R.; Canales-Vázquez, J.; Castro-García, M.; Villas, M.V.; Mansilla Legorburo, F.; Sabater, S. Radiomics of CT features may be nonreproducible and redundant: Influence of CT acquisition parameters. *Radiology* **2018**, *288*, 407–415. https://doi.org/10.1148/radiol.2018172361.
18. Radiomics Image Biomarker Standardisation Initiative (IBSI). Available online: https://ibsi-radiomics.org (accessed on 5 May 2026).
19. Radiological Society of North America (RSNA). Radiomics. Available online: https://pubs.rsna.org/ (accessed on 5 May 2026).
20. Narrative Review checklist. Available online: https://legacyfileshare.elsevier.com/promis_misc/ANDJ%20Narrative%20Review%20Checklist.pdf (accessed on 5 May 2026).
21. Fuster-Matanzo, A.; Picó-Peris, A.; Bellvís-Bataller, F.; Jimenez-Pastor, A.; Weiss, G.J.; Martí-Bonmatí; L; Lázaro Sánchez, A.; Bazaga, D.; Banna, G.L.; Addeo, A.; et al. Prediction of oncogene mutation status in non-small cell lung cancer: A systematic review and meta-analysis with a special focus on artificial intelligence-based methods. *Eur. Radiol.* **2026**, *36*, 2157–2185. https://doi.org/10.1007/s00330-025-11962-x.
22. Zhang, J.; Lin, J.; Wang, J.; Liang, Y.; Gao, C.; Zheng, H. Application of dual-energy computed tomography combined with radiomics in the clinical diagnosis of lung cancer: A systematic review and meta-analysis. *J. Thorac. Dis.* **2026**, *18*, 145. https://doi.org/10.21037/jtd-2025-1-2449.
23. Chen, H.; Fan, B.; Yuan, M.; Wang, D.; Qiao, C.; Qiu, N.; Quan, X.; Hou, W. CT-based radiomics in predicting the efficacy of preoperative neoadjuvant chemoimmunotherapy for non-small cell lung cancer: A systematic review and meta-analysis. *Front. Immunol.* **2026**, *17*, 1753166. https://doi.org/10.3389/fimmu.2026.1753166.
24. Nayak, K.; Kadavigere, R.; Pendem, S.; Mane, P.R.; Sampathila, N.; Pattath Sankaran, P.; Siddeshappa, N. Diagnostic Accuracy of Artificial Intelligence Models for Differentiation of Squamous Cell Carcinoma and Adenocarcinoma of Lung-A Systematic Review. *Diagnostics* **2026**, *16*, 500. https://doi.org/10.3390/diagnostics16030500.
25. Chang, C.W.; Wang, T.; Qiu, R.L.J.; Li, X.; Cammin, J.; Yang, K.; Hu, Y.H.; Ren, L.; Xia, P.; Sawant, A.; et al. Patient outcome prognosis for external beam radiation therapy using CBCT-based radiomics: A systematic review. *Biomed. Phys. Eng. Express* **2026**, *12*, 012002. https://doi.org/10.1088/2057-1976/ae308b.
26. Sahrai, H.; Behnood, J.; Baradaran, M.; Khalaji, A.; Norouzi, A.; Shojaeshafiei, F.; Seyed Ebrahimi, S.M.; Mohammadzadeh, S.; HajiEsmailpoor, Z.; Shahidi, R. Diagnostic accuracy of CT-based radiomics models in differentiating lung cancer from tuberculosis in pulmonary lesions: A systematic review and meta-analysis. *BMC Cancer* **2025**, *26*, 111. https://doi.org/10.1186/s12885-025-15446-5.
27. Salimi, M.; Vadipour, P.; Khosravi, A.; Salimi, B.; Mabani, M.; Rostami, P.; Seifi, S. CT-Based Radiomics for Predicting PD-L1 Expression in Non-small Cell Lung Cancer: A Systematic Review and Meta-analysis. *Acad. Radiol.* **2025**, *32*, 6913–6928. https://doi.org/10.1016/j.acra.2025.05.024.

28. Chen, L.; Lan, X.; Huang, Y.; Tao, J.; Huang, X.; Su, Y.; Liu, D.; Fang, X.; Zhang, J. CT-based radiomics models for predicting spread through air space in lung cancer: A systematic review and meta-analysis. *Eur. J. Radiol.* **2025**, *190*, 112249. https://doi.org/10.1016/j.ejrad.2025.112249.
29. Zhang, Y.R.; Lu, Y.H.; Lin, C.M.; Ku, J.W. Pretreatment CT Texture Analysis for Predicting Survival Outcomes in Advanced Nonsmall Cell Lung Cancer Patients Receiving Immunotherapy: A Systematic Review and Meta-Analysis. *Thorac. Cancer* **2025**, *16*, e70144. https://doi.org/10.1111/1759-7714.70144.
30. Liu, H.; Shu, P.; Zhao, L.; Chunfeng, L.; Lun, L. Machine learning approaches for EGFR mutation status prediction in NSCLC: An updated systematic review. *Front. Oncol.* **2025**, *15*, 1576461. https://doi.org/10.3389/fonc.2025.1576461.
31. Chen, J.; Zhang, X.; Xu, C.; Liu, K. Diagnostic performance of radiomics analysis for pulmonary cancer airway spread: A systematic review and meta-analysis. *Diagn. Interv. Radiol.* **2025**, *31*, 215–225. https://doi.org/10.4274/dir.2024.242852.
32. Shahidi, R.; Hassannejad, E.; Baradaran, M.; Klontzas, M.E.; ShahirEftekhar, M.; Shojaeshafiei, F.; HajiEsmailPoor, Z.; Chong, W.; Broomand, N.; Alizadeh, M.; et al. Diagnostic performance of radiomics in prediction of Ki-67 index status in non-small cell lung cancer: A systematic review and meta-analysis. *J. Med. Imaging Radiat. Sci.* **2024**, *55*, 101746. https://doi.org/10.1016/j.jmir.2024.101746.
33. Jiang, Y.; Gao, C.; Shao, Y.; Lou, X.; Hua, M.; Lin, J.; Wu, L.; Gao, C. The prognostic value of radiogenomics using CT in patients with lung cancer: A systematic review. *Insights Into Imaging* **2024**, *15*, 259. https://doi.org/10.1186/s13244-024-01831-4.
34. Tran, K.; Ginzburg, D.; Hong, W.; Attenberger, U.; Ko, H.S. Post-radiotherapy stage III/IV non-small cell lung cancer radiomics research: A systematic review and comparison of CLEAR and RQS frameworks. *Eur. Radiol.* **2024**, *34*, 6527–6543. https://doi.org/10.1007/s00330-024-10736-1.
35. Jia, X.; Wang, Y.; Zhang, H.; Sun, D. Current status and quality of prognosis prediction models of non-small cell lung cancer constructed using computed tomography (CT)-based radiomics: A systematic review and radiomics quality score 2.0 assessment. *Quant. Imaging Med. Surg.* **2024**, *14*, 6978–6989. https://doi.org/10.21037/qims-24-22.
36. Cheng, D.O.; Khaw, C.R.; McCabe, J.; Pennycuick, A.; Nair, A.; Moore, D.A.; Janes, S.M.; Jacob, J. Predicting histopathological features of aggressiveness in lung cancer using CT radiomics: A systematic review. *Clin. Radiol.* **2024**, *79*, 681–689. https://doi.org/10.1016/j.crad.2024.04.022.
37. Chen, J.; Chen, A.; Yang, S.; Liu, J.; Xie, C.; Jiang, H. Accuracy of machine learning in preoperative identification of genetic mutation status in lung cancer: A systematic review and meta-analysis. *Radiother. Oncol. J. Eur. Soc. Ther. Radiol. Oncol.* **2024**, *196*, 110325. https://doi.org/10.1016/j.radonc.2024.110325.
38. Luo, X.; Zheng, R.; Zhang, J.; He, J.; Luo, W.; Jiang, Z.; Li, Q. CT-based radiomics for predicting Ki-67 expression in lung cancer: A systematic review and meta-analysis. *Front. Oncol.* **2024**, *14*, 1329801. https://doi.org/10.3389/fonc.2024.1329801.
39. Nguyen, H.S.; Ho, D.K.N.; Nguyen, N.N.; Tran, H.M.; Tam, K.W.; Le, N.Q.K. Predicting EGFR Mutation Status in Non-Small Cell Lung Cancer Using Artificial Intelligence: A Systematic Review and Meta-Analysis. *Acad. Radiol.* **2024**, *31*, 660–683. https://doi.org/10.1016/j.acra.2023.03.040.
40. Chiu, H.Y.; Wang, T.W.; Hsu, M.S.; Chao, H.S.; Liao, C.Y.; Lu, C.F.; Wu, Y.T.; Chen, Y.M. Progress in Serial Imaging for Prognostic Stratification of Lung Cancer Patients Receiving Immunotherapy: A Systematic Review and Meta-Analysis. *Cancers* **2024**, *16*, 615. https://doi.org/10.3390/cancers16030615.
41. Shi, L.; Sheng, M.; Wei, Z.; Liu, L.; Zhao, J. CT-Based Radiomics Predicts the Malignancy of Pulmonary Nodules: A Systematic Review and Meta-Analysis. *Acad. Radiol.* **2023**, *30*, 3064–3075. https://doi.org/10.1016/j.acra.2023.05.026.
42. Felfli, M.; Liu, Y.; Zerka, F.; Voyton, C.; Thinnes, A.; Jacques, S.; Iannessi, A.; Bodard, S. Systematic Review, Meta-Analysis and Radiomics Quality Score Assessment of CT Radiomics-Based Models Predicting Tumor EGFR Mutation Status in Patients with Non-Small-Cell Lung Cancer. *Int. J. Mol. Sci.* **2023**, *24*, 11433. https://doi.org/10.3390/ijms241411433.
43. Wang, T.W.; Hsu, M.S.; Lin, Y.H.; Chiu, H.Y.; Chao, H.S.; Liao, C.Y.; Lu, C.F.; Wu, Y.T.; Huang, J.W.; Chen, Y.M. Application of Radiomics in Prognosing Lung Cancer Treated with Epidermal Growth Factor Receptor Tyrosine Kinase Inhibitors: A Systematic Review and Meta-Analysis. *Cancers* **2023**, *15*, 3542. https://doi.org/10.3390/cancers15143542.
44. Wu, L.; Lou, X.; Kong, N.; Xu, M.; Gao, C. Can quantitative peritumoral CT radiomics features predict the prognosis of patients with non-small cell lung cancer? A systematic review. *Eur. Radiol.* **2023**, *33*, 2105–2117. https://doi.org/10.1007/s00330-022-09174-8.
45. Lee, K.; Le, T.; Hau, E.; Hanna, G.G.; Gee, H.; Vinod, S.; Dammak, S.; Palma, D.; Ong, A.; Yeghiaian-Alvandi, R.; et al. A Systematic Review Into the Radiologic Features Predicting Local Recurrence After Stereotactic Ablative Body Radiotherapy (SABR) in Patients with Non-Small Cell Lung Cancer (NSCLC). *Int. J. Radiat. Oncol. Biol. Phys.* **2022**, *113*, 40–59. https://doi.org/10.1016/j.ijrobp.2021.11.027.

46. Zhang, C.; de AFFonseca, L.; Shi, Z.; Zhu, C.; Dekker, A.; Bermejo, I.; Wee, L. Systematic review of radiomic biomarkers for predicting immune checkpoint inhibitor treatment outcomes. *Methods* **2021**, *188*, 61–72. https://doi.org/10.1016/j.ymeth.2020.11.005.
47. van Laar, M.; van Amsterdam, W.A.C.; van Lindert, A.S.R.; de Jong, P.A.; Verhoeff, J.J.C. Prognostic factors for overall survival of stage III non-small cell lung cancer patients on computed tomography: A systematic review and meta-analysis. *Radiother. Oncol. J. Eur. Soc. Ther. Radiol. Oncol.* **2020**, *151*, 152–175. https://doi.org/10.1016/j.radonc.2020.07.030.
48. Sukhera, J. Narrative Reviews: Flexible, Rigorous, and Practical. *J. Grad. Med Educ.* **2022**, *14*, 414–417. https://doi.org/10.4300/JGME-D-22-00480.1. PMID: 35991099; PMCID: PMC9380636.
49. MW Editing. Narrative Review vs. Systematic Review. Available online: https://www.mwediting.com/narrative-review-vs-systematic-review/ (accessed on 5 May 2026).
50. MD Anderson Cancer Center. Narrative Reviews. Available online: https://www3.mdanderson.org/library/education/quick-help/writing/narrative-review.html (accessed on 5 May 2026).
51. Kunkyab, T.; Hyde, D.; Mou, B.; Abrina, J.; Clark, H.; Javanmardi, A.; Baker, S. Development of Radiomics Models to Predict Progression-Free Survival and Early Polymetastatic Progression in Patients with Lung Oligometastases Treated on the Single-Arm Phase II Stereotactic Ablative Radiotherapy-5 Trial. *Clin. Oncol.* **2026**, *52*, 104079. https://doi.org/10.1016/j.clon.2026.104079.
52. Italiano, A.; Gautier, O.; Dupont, J.; Assi, T.; Dawi, L.; Lawrance, L.; Lassau, N. The correlation of liquid biopsy genomic data to radiomics in colon, pancreatic, lung and prostatic cancer patients. *Eur. J. Cancer* **2025**, *226*, 115609. https://doi.org/10.1016/j.ejca.2025.115609.
53. Zhou, J.; Wen, Y.; Ding, R.; Liu, J.; Fang, H.; Li, X.; Wan, Q. Radiomics signature based on diffusion data for differentiation between benign and malignant solitary pulmonary lesions. *Cancer Imaging* **2024**, *24*, 14. https://doi.org/10.1186/s40644-024-00660-4.
54. Liu, F.; Xiang, Z.; Li, Q.; Fang, X.; Zhou, J.; Yang, X.; Yang, Q. 18F-FDG PET/CT-based radiomics model for predicting pathological differentiation in NSCLC: Multicentre study. *Clin. Radiol.* **2024**, *79*, e147–e155. https://doi.org/10.1016/j.crad.2023.09.017.
55. Ren, C.; Zhang, F.; Zhang, J.; Song, S.; Sun, Y.; Cheng, J. Clinico-biological-radiomics (CBR) machine learning for FDG-PET lymph node classification in lung cancer. *Eur. J. Med. Res.* **2023**, *28*, 554. https://doi.org/10.1186/s40001-023-01497-6.
56. Zhu, Z.C.; Chen, M.J.; Song, L.; Wang, J.H.; Hu, G.; Han, W.; Tan, W.X.; Zhou, Z.; Sui, X.; Song, W.; et al. CT-based radiomic score predicts immunotherapy response in NSCLC. *Zhongguo Yi Xue Ke Xue Yuan Xue Bao* **2023**, *45*, 794–802. https://doi.org/10.3881/j.issn.1000-503X.15705.
57. Hongwei, S.; Xinzhong, H.; Huiqin, X.; Shuqin, X.; Ruonan, W.; Li, L.; Jianzhong, C.; Sijin, L. Standard deviation of CT radiomic features among malignancies in each individual: prognostic ability in lung cancer patients. J Cancer Res Clin Oncol 2023, 149, 7165–7173. https://doi.org/10.1007/s00432-023-04649-7.
58. Lin, J.; Yu, Y.; Zhang, X.; Wang, Z.; Li, S. Classification of Histological Types and Stages in Non-small Cell Lung Cancer Using Radiomic Features Based on CT Images. *J. Digit. Imaging* **2023**, *36*, 1029–1037. https://doi.org/10.1007/s10278-023-00792-2.
59. Dercle, L.; Fronheiser, M.; Rizvi, N.A.; Hellmann, M.D.; Maier, S.; Hayes, W.; Yang, H.; Guo, P.; Fojo, T.; Schwartz, L.H.; et al. Baseline Radiomic Signature to Estimate Overall Survival in Patients With NSCLC. *J. Thorac. Oncol*. **2023**, *18*, 587–598. https://doi.org/10.1016/j.jtho.2022.12.019.
60. Zhang, H.; Liao, M.; Guo, Q.; Chen, J.; Wang, S.; Liu, S.; Xiao, F. Predicting N2 lymph node metastasis in presurgical stage I-II non-small cell lung cancer using multiview radiomics and deep learning method. *Med. Phys.* **2023**, *50*, 2049–2060. https://doi.org/10.1002/mp.16177.
61. Liu, C.; Zhao, W.; Xie, J.; Lin, H.; Hu, X.; Li, C.; Shang, Y.; Wang, Y.; Jiang, Y.; Ding, M.; et al. Development and validation of a radiomics-based nomogram for predicting a major pathological response to neoadjuvant immunochemotherapy for patients with potentially resectable non-small cell lung cancer. *Front. Immunol.* **2023**, *14*, 1115291. https://doi.org/10.3389/fimmu.2023.1115291.
62. Sun, Y.; Ma, Z.; Zhao, W.; Jin, L.; Gao, P.; Wang, K.; Huang, X.; Duan, S.; Li, M. Computed tomography radiomics in growth prediction of pulmonary ground-glass nodules. *Eur. J. Radiol.* **2023**, *159*, 110684. https://doi.org/10.1016/j.ejrad.2022.110684.
63. Chen, Q.; Shao, J.; Xue, T.; Peng, H.; Li, M.; Duan, S.; Feng, F. Intratumoral and peritumoral radiomics nomograms for the preoperative prediction of lymphovascular invasion and overall survival in non-small cell lung cancer. *Eur. Radiol.* **2023**, *33*, 947–958. https://doi.org/10.1007/s00330-022-09109-3.
64. Zhang, J.; Hao, L.; Li, M.; Xu, Q.; Shi, G. CT Radiomics Combined With Clinicopathological Features to Predict Invasive Mucinous Adenocarcinoma in Patients With Lung Adenocarcinoma. *Technol. Cancer Res. Treat.* **2023**, *22*, 15330338231174306. https://doi.org/10.1177/15330338231174306.

65. Yu, Y.; Tan, J.; Yang, Y.; Zhang, B.; Yao, X.; Sang, S.; Deng, S. The Differential Diagnostic Value of Radiomics Signatures Between Single-Nodule Pulmonary Metastases and Second Primary Lung Cancer in Patients with Colorectal Cancer. *Technol. Cancer Res. Treat.* **2023**, *22*, 15330338231175735. https://doi.org/10.1177/15330338231175735.
66. Chen, N.-B.; Xiong, M.; Zhou, R.; Zhou, Y.; Qiu, B.; Luo, Y.-F.; Zhou, S.; Chu, C.; Li, Q.-W.; Wang, B.; et al. CT radiomics-based long-term survival prediction for locally advanced non-small cell lung cancer patients treated with concurrent chemoradiotherapy using features from tumor and tumor organismal environment. *Radiat. Oncol.* **2022**, *17*, 184. https://doi.org/10.1186/s13014-022-02136-w.
67. Jiang, Y.; Wang, Y.; Fu, S.; Chen, T.; Zhou, Y.; Zhang, X.; Chen, C.; He, L.; Du, W.; Li, H.; et al. A CT-based radiomics model to predict subsequent brain metastasis in patients with ALK-rearranged non–small cell lung cancer undergoing crizotinib treatment. *Thorac. Cancer* **2022**, *13*, 1558–1569. https://doi.org/10.1111/1759-7714.14386.
68. Xue, L.M.; Li, Y.; Zhang, Y.; Wang, S.C.; Zhang, R.Y.; Ye, J.D.; Yu, H.; Qiang, J.W. A predictive nomogram for two-year growth of CT-indeterminate small pulmonary nodules. *Eur. Radiol.* **2022**, *32*, 2672–2682. https://doi.org/10.1007/s00330-021-08343-5.
69. Yao, W.; Liao, Y.; Li, X.; Zhang, F.; Zhang, H.; Hu, B.; Wang, X.; Li, L.; Xiao, M. Noninvasive Method for Predicting the Expression of Ki67 and Prognosis in Non-Small-Cell Lung Cancer Patients: Radiomics. *J. Healthc. Eng.* **2022**, *2022*, 7761589. https://doi.org/10.1155/2022/7761589.
70. Huang, L.; Lin, W.; Xie, D.; Yu, Y.; Cao, H.; Liao, G.; Wu, S.; Yao, L.; Wang, Z.; Wang, M.; et al. Development and validation of a preoperative CT-based radiomic nomogram to predict pathology invasiveness in patients with a solitary pulmonary nodule: a machine learning approach, multicenter, diagnostic study. *Eur. Radiol.* **2022**, *32*, 1983–1996. https://doi.org/10.1007/s00330-021-08268-z.
71. Zwanenburg, A.; Vallières, M.; Abdalah, M.A.; Aerts, H.J.W.L.; Andrearczyk, V.; Apte, A.; Ashrafinia, S.; Bakas, S.; Beukinga, R.J.; Boellaard, R.; et al. The Image Biomarker Standardization Initiative: Standardized Quantitative Radiomics for High-Throughput Image-based Phenotyping. *Radiology* **2020**, *295*, 328–338. https://doi.org/10.1148/radiol.2020191145. PMID: 32154773; PMCID: PMC7193906.
72. Shukla-Dave, A.; Obuchowski, N.A.; Chenevert, T.L.; Jambawalikar, S.; Schwartz, L.H.; Malyarenko, D.; Huang, W.; Noworolski, S.M.; Young, R.J.; Shiroishi, M.S.; et al. Quantitative imaging biomarkers alliance (QIBA) recommendations for improved precision of DWI and DCE-MRI derived biomarkers in multicenter oncology trials. *J. Magn. Reason. Imaging* **2019**, *49*, e101–e121. https://doi.org/10.1002/jmri.26518. PMID: 30451345; PMCID: PMC6526078.
73. Collins, G.S.; Reitsma, J.B.; Altman, D.G.; Moons, K.G. Transparent reporting of a multivariable prediction model for individual prognosis or diagnosis (TRIPOD): The TRIPOD statement. *BMJ* **2015**, *350*, g7594. https://doi.org/10.1136/bmj.g7594. PMID: 25569120.
74. Hatt, M.; Krizsan, A.K.; Rahmim, A.; Bradshaw, T.J.; Costa, P.F.; Forgacs, A.; Seifert, R.; Zwanenburg, A.; El Naqa, I.; Kinahan, P.E.; et al. Joint EANM/SNMMI guideline on radiomics in nuclear medicine: Jointly supported by the EANM Physics Committee and the SNMMI Physics, Instrumentation and Data Sciences Council. *Eur. J. Nucl. Med. Mol. Imaging* **2023**, *50*, 352–375. https://doi.org/10.1007/s00259-022-06001-6. PMID: 36326868; PMCID: PMC9816255.
75. Food and Drug Administration (FDA). Software as a Medical Device (SaMD). Available online: https://www.fda.gov/medical-devices/digital-health-center-excellence/software-medical-device-samd (accessed on 5 May 2026).
76. European Union. Regulation (EU) 2024/1689 of the European Parliament and of the Council (Artificial Intelligence Act). Available online: https://eur-lex.europa.eu/eli/reg/2024/1689/oj (accessed on 5 May 2026).
77. Ethics and Governance of Artificial Intelligence for Health. Available online: https://www.who.int/publications/i/item/9789240029200 (accessed on 5 May 2026).
78. de Koning, H.J.; Van Der Aalst, C.M.; De Jong, P.A.; Scholten, E.T.; Nackaerts, K.; Heuvelmans, M.A.; Lammers, J.-W.J.; Weenink, C.; Yousaf-Khan, U.; Horeweg, N.; et al. Reduced Lung-Cancer Mortality with Volume CT Screening in a Randomized Trial. *N. Engl. J. Med.* **2020**, *382*, 503–513. https://doi.org/10.1056/NEJMoa1911793. PMID: 31995683.

Disclaimer/Publisher's Note: The statements, opinions and data contained in all publications are solely those of the individual author(s) and contributor(s) and not of MDPI and/or the editor(s). MDPI and/or the editor(s) disclaim responsibility for any injury to people or property resulting from any ideas, methods, instructions or products referred to in the content.
